# Targeting nucleotide metabolic pathways in colorectal cancer by integrating scRNA-seq, spatial transcriptome, and bulk RNA-seq data

**DOI:** 10.1007/s10142-024-01356-5

**Published:** 2024-04-10

**Authors:** Songyun Zhao, Pengpeng Zhang, Sen Niu, Jiaheng Xie, Yuankun Liu, Yuan Liu, Ning Zhao, Chao Cheng, Peihua Lu

**Affiliations:** 1 Wuxi Medical Center of Nanjing Medical University, Wuxi, China; 2 Department of Neurosurgery, Affiliated Wuxi People’s Hospital of Nanjing Medical University, Wuxi, China; 3 Department of Lung Cancer Surgery, Tianjin Medical University Cancer Institute and Hospital, Tianjin, China; 4 Department of General Surgery, Affiliated Wuxi People’s Hospital of Nanjing Medical University, Wuxi, China; 5 Department of Plastic Surgery, Xiangya Hospital, Central South University, Changsha, China; 6 Department of Clinical Research Center, Affiliated Wuxi People’s Hospital of Nanjing Medical University, Wuxi, China

**Keywords:** Nucleotide metabolism, Colorectal cancer, scRNA-seq, stRNA-seq, Prognostic model, Immunotherapy

## Abstract

**Background:**

Colorectal cancer is a malignant tumor of the digestive system originating from abnormal cell proliferation in the colon or rectum, often leading to gastrointestinal symptoms and severe health issues. Nucleotide metabolism, which encompasses the synthesis of DNA and RNA, is a pivotal cellular biochemical process that significantly impacts both the progression and therapeutic strategies of colorectal cancer

**Methods:**

For single-cell RNA sequencing (scRNA-seq), five functions were employed to calculate scores related to nucleotide metabolism. Cell developmental trajectory analysis and intercellular interaction analysis were utilized to explore the metabolic characteristics and communication patterns of different epithelial cells. These findings were further validated using spatial transcriptome RNA sequencing (stRNA-seq). A risk model was constructed using expression profile data from TCGA and GEO cohorts to optimize clinical decision-making. Key nucleotide metabolism-related genes (NMRGs) were functionally validated by further in vitro experiments.

**Results:**

In both scRNA-seq and stRNA-seq, colorectal cancer (CRC) exhibited unique cellular heterogeneity, with myeloid cells and epithelial cells in tumor samples displaying higher nucleotide metabolism scores. Analysis of intercellular communication revealed enhanced signaling pathways and ligand-receptor interactions between epithelial cells with high nucleotide metabolism and fibroblasts. Spatial transcriptome sequencing confirmed elevated nucleotide metabolism states in the core region of tumor tissue. After identifying differentially expressed NMRGs in epithelial cells, a risk prognostic model based on four genes effectively predicted overall survival and immunotherapy outcomes in patients. High-risk group patients exhibited an immunosuppressive microenvironment and relatively poorer prognosis and responses to chemotherapy and immunotherapy. Finally, based on data analysis and a series of cellular functional experiments, ACOX1 and CPT2 were identified as novel therapeutic targets for CRC.

**Conclusion:**

In this study, a comprehensive analysis of NMRGs in CRC was conducted using a combination of single-cell sequencing, spatial transcriptome sequencing, and high-throughput data. The prognostic model constructed with NMRGs shows potential as a standalone prognostic marker for colorectal cancer patients and may significantly influence the development of personalized treatment approaches for CRC.

**Supplementary Information:**

The online version contains supplementary material available at 10.1007/s10142-024-01356-5.

## Introduction

Colorectal cancer (CRC) stands as a prominent global health concern, responsible for the highest number of cancer-related fatalities across the world. It ushers in over one million fresh cases annually. In the United States alone, the year 2021 witnessed an estimated 104,000 individuals newly diagnosed with colorectal cancer, accompanied by a sobering 53,000 new cases culminating in mortality. These statistics collectively position CRC as the third most frequently diagnosed cancer and the primary contributor to cancer-related fatalities (Sung et al. 2021). The 5-year relative survival rate of colorectal cancer patients is only 64%, and although existing treatments including chemotherapy and surgery have improved survival, however, distant metastasis and drug resistance remain the main reasons for the poor prognostic outcome of colorectal cancer patients (Chen et al. 2020). Colorectal adenocarcinoma is the main pathologic type of CRC, which is associated with age, dietary habits, and genetic disorders, and is significantly heterogeneous (Barresi et al. 2015). The exact molecular mechanism of carcinogenesis in colorectal cancer is currently uncertain, and timely diagnosis and treatment using reliable biomarkers can significantly improve survival (van der Geest et al. 2015).

A fundamental characteristic of cancer cells is their altered metabolism, which exhibits numerous distinctions from the metabolic patterns observed in normal cells. This phenomenon, known as metabolic reprogramming, plays a pivotal role in influencing the initiation and advancement of tumors (Zhang et al. 2021a). One of the critical facets of metabolic reprogramming in cancer revolves around the production and utilization of nucleotide triphosphates. This metabolic shift is prevalent across various cancer types and genetic backgrounds. Importantly, many aggressive behaviors exhibited by cancer cells, such as unchecked proliferation, resistance to chemotherapy, evasion of immune surveillance, and the ability to metastasize, rely heavily on the upregulated nucleotide metabolism (Mullen and Singh 2023). The heightened demand for nucleotides is particularly pronounced in rapidly dividing cancer cells, which necessitate increased nitrogen utilization for the synthesis of essential nitrogen-containing molecules, including nucleotides. Disruptions in nucleotide metabolism within tumor tissues can thus fuel tumor growth and potentially impact interactions with the host immune system, thereby influencing the effectiveness of immunotherapies (Wu et al. 2022a).

Within the complex milieu of the tumor microenvironment, aberrations in nucleotide metabolism can significantly modify normal immune responses. This underscores the potential value of targeting nucleotide metabolism as a strategy to bolster immunotherapeutic interventions (Ishii and Akira 2008). Numerous investigations have demonstrated that tumor cells, in their quest for survival, can adapt their nucleotide metabolic pathways as they evolve drug resistance, thereby evading the intended effects of therapy (Zou et al. 2023). Conversely, irregularities in nucleotide metabolism can also serve as vulnerable points for therapeutic resistance. Through strategic interventions aimed at disrupting nucleotide metabolic pathways, it becomes possible to reverse the drug-resistant state of tumor cells and amplify the effectiveness of therapeutic agents (Mullen and Singh 2023; Tyagi et al. 2022). Notably, in preclinical animal models, therapies specifically aimed at modulating nucleotide metabolism have yielded promising outcomes (Kohnken et al. 2015).

Even though single-cell sequencing has advanced tumor metabolism studies, our knowledge of nucleotide metabolism in colorectal cancer is still restricted (Zhao et al. 2023). Colorectal cancer is known for its complex cellular composition and microenvironment, making nucleotide metabolism effects intricate. To explore the role of nucleotide metabolism-related genes (NMRGs), we conducted single-cell and spatial transcriptome sequencing to characterize the NMRG microenvironment in colorectal cancer. We also developed a prognostic model using bulk RNA sequencing, aiding clinicians in treatment decisions. We identified two new immunotherapy targets, ACOX1 and CPT2, validating them through bioinformatics and cellular experiments. These studies illuminate the regulation of nucleotide metabolism, providing a theoretical foundation for the development of future targeted and immunotherapeutic strategies.

## Methods

### Original research data source

The TCGA-COADREAD mRNA-seq data, clinical data, and single nucleotide mutation data were obtained from the UCSC Xena website (https://xenabrowser.net/). Additionally, RNA-seq data for colorectal cancer patients, along with corresponding clinical information from the GSE17538 and GSE39582 datasets, were retrieved from the Gene Expression Omnibus (GEO) database (http://www.ncbi.nlm.nih.gov/geo/). The TCGA cohort comprised 584 tumor patients with available survival information, whereas the GSE17538 and GSE39582 cohorts, utilized as validation models, consisted of 232 and 579 CRC patients with survival information, respectively. In the TCGA dataset, gene expression profiles were quantified using the transcript per million (TPM) estimation and subsequently transformed using a log2-based approach. The gene expression datasets underwent batch calibration and integration processes utilizing the “limma” and “sva” R packages (Leek et al. 2012). For single-cell RNA sequencing data of CRC, we accessed the GSE166555 cohort from TISCH (http://tisch.comp-genomics.org/), comprising a total of 66,050 cells from both tumor and normal tissues (Uhlitz et al. 2021). Spatial transcriptome data for primary CRC were downloaded from The National Omics Data Encyclopedia (https://www.biosino.org/node/project/detail/OEP001756) (Wu et al. 2022b). A total of 882 mRNAs with relevance scores exceeding 10 were identified by querying the GeneCard database (https://www.genecards.org/) using the keyword “Nucleotide metabolism” (Supplementary table 1) (Rebhan et al. 1997).

### Processing of scRNA-seq data and calculation of nucleotide metabolism scores

The single-cell RNA sequencing (scRNA-seq) data were preprocessed using the “Seurat” R package (Hao et al. 2021). The “PercentageFeatureSet” function was applied to assess the proportion of mitochondrial genes in the dataset. To ensure data quality and integrity, only genes expressed in a minimum of three cells were retained. Moreover, each cell was required to express more than 200 genes but fewer than 10,000 genes, with mitochondrial content constituting less than 20%. Subsequently, normalization of the scRNA-seq data was performed using the “NormalizeData” function.

Following normalization, the data were transformed into Seurat objects, and the “FindVariableFeatures” function was utilized to identify the top 2000 highly variable genes. Subsequently, the “RunPCA” tool was employed for scaling and principal component analysis on this set of highly variable genes. Dimensionality reduction and visualization in 2D coordinates were achieved through Shared Nearest Neighbor (SNN) modular optimization and the t-distributed Stochastic Neighborhood Embedding (t-SNE) clustering algorithm. To identify marker genes specifically expressed in each cluster, Wilcoxon tests were conducted using the “FindAllMarkers” and “FindMarkers” algorithms, comparing various cell types. Additionally, various cell subgroups were annotated based on marker genes, incorporating insights from the original article and annotations available in the TISCH database.

We analyzed 882 genes based on their expression profiles associated with nucleotide metabolism. To assess the enrichment scores of colorectal cancer single-cell sequencing data, we employed five widely used algorithms: “AddModuleScore”, “AUCell”, “ssGSEA”, “singscore”, and “UCell”. “AddModuleScore” is an algorithm integrated into the SingleR package (Tirosh et al. 2016), which computes enrichment scores by determining the average expression value of all genes within a given gene set and then scoring the gene sets based on this average value. This method involves calculating the average expression value for all genes in the gene set and partitioning the expression matrix into segments based on these average values. Control genes are randomly selected as background values from each partitioned segment. Single-sample gene set enrichment analysis, commonly referred to as “ssGSEA”, is utilized to evaluate the extent of enrichment of a specific gene set within an individual sample or cell. “AUCell” is a tool in R for assessing the enrichment of a given set of genes in gene expression data from a single sample, aiding in the understanding of biological processes and disease mechanisms. The “UCell” method is employed for unsupervised cell type identification. It categorizes the cell type of specific cells without relying on past information or labels (Andreatta and Carmona 2021). “singscore” quantifies the activity level of a specific biological function or process within a single sample or cell, serving as a method for evaluating the cellular state.

We constructed single-cell pseudotime trajectories for epithelial cells utilizing the “Monocle” R package (Borcherding et al. 2019). To explore crucial genes implicated in cell development along these trajectories, we applied the BEAM algorithm. Furthermore, we leveraged the functionalities of the “CellCall” R software package, a valuable tool for unveiling intricate signaling profiles by integrating both intracellular and intercellular events (Zhang et al. 2021b).

### Processing CRC spatial transcriptome sequencing data and inferring cellular spatial interactions

Spatial transcriptome data analysis was conducted using the R package Seurat. This involved normalizing unique molecular identifier (UMI) counts, scaling the data, and identifying the most variable features using “SCTransform”. Subsequently, downscaling and unsupervised cluster analysis were performed using “RunPCA”. For cluster analysis, default parameters were utilized, focusing on the 30 most significant principal components. Additionally, the “SpatialFeaturePlot” function was employed for subgroup and gene visualization. The “scMetabolism” R package serves as a valuable tool for single-cell metabolic analysis. Its primary function is to quantify and visualize metabolic activities at single-cell resolution. Researchers can utilize “scMetabolism” to investigate metabolic differences between different cells, identify metabolic signatures associated with specific cell types or conditions, and visualize metabolic data (Wu et al. 2022b). Scanpy is a Python toolkit designed for single-cell RNA sequencing data analysis. It offers a wide range of features including data preprocessing, visualization, clustering, and differential gene analysis for studying gene expression at the single-cell level. In contrast, stLearn, based on Scanpy, focuses specifically on spatial transcriptome data analysis. It combines gene expression and image information to understand the relationship between cell location in a tissue and gene expression patterns. RCTD is a computational method aimed at parsing transcriptome data from different cell types or populations within complex biological samples. Its primary objective is to extract the gene expression profiles of each cell type or population from mixed transcriptomic data, facilitating the understanding of cellular composition, function, and interactions in biological research. MISTy is a machine-learning-based interpretable multi-view framework tailored for parsing highly multiplexed intercellular relationships in data. It provides researchers with a means to analyze spatial transcriptomic data without the need for cell type annotations, aiding in the comprehension of patterns and mechanisms of cell interactions (Tanevski et al. 2022). MISTy has been implemented as an R package called mistyR, accompanied by detailed documentation and instructions (https://saezlab.github.io/mistyR/).

### Construction of prognostic models and calculation of risk scores

The TCGA cohort was utilized as the training set, while the GSE17538 and GSE39582 datasets were combined to form the GEO cohort, serving as the validation set. Based on the differential genes obtained from single-cell sequencing, univariate Cox regression analysis was performed using the “survival” software R package to identify prognostic genes with statistically significant *p* < 0.05. Subsequently, lasso and multifactorial stepwise regression analyses were conducted to further evaluate genes and risk variables highly associated with prognosis.

Each CRC patient was assigned a risk score based on the coefficients derived from multivariate analysis. The TCGA and GEO cohorts were then divided into high-risk and low-risk groups based on the median risk score. The computation of the risk score followed the equation:

$$\textrm{Riskscore}={\textrm{h}}_0\left(\textrm{t}\right)\ast \exp \left({\textrm{a}}_1\ast {\textrm{x}}_1+{\textrm{a}}_2\ast {\textrm{x}}_2+\dots +{\textrm{a}}_{\textrm{n}}\ast {\textrm{x}}_{\textrm{n}}\right)$$

Here, h_0_(t) represents the baseline risk function, indicating the risk when all independent variables are 0, and exp denotes the increment of risk at time t over what it would be if the independent variables were 0. The coefficients a_1_, a_2_, ..., a_n_ correspond to the regression coefficients of the Cox model, while x_1_, x_2_, ..., x_n_ represent the respective independent variables (gene expression values), observed at time t. The Cox model forms the basis for this computation.

Furthermore, survival curves were generated using the Kaplan-Meier method to predict the prognosis of CRC patients, with the log-rank test employed to determine statistical significance. The predictive model's validity was assessed using receiver operating characteristic (ROC) curves, with performance deemed satisfactory when the area under the curve (AUC) value exceeded 0.65.

### Nomogram construction and raw data for mutation analysis

To compute the probability of overall survival (OS) at 1, 3, and 5 years, we constructed a column-line graph incorporating risk score, age, and clinical stage as independent prognostic factors. Additionally, consistency index analysis and decision curve analysis (DCA) were conducted to further evaluate the utility of the column chart in conjunction with clinical characteristics alone.

Somatic mutations observed in high-risk and low-risk cohorts of colorectal cancer were calculated using the “maftools” R package. Mutation Annotation Format (MAF) files were generated using data sourced from the TCGA database. Furthermore, we explored the relationship between risk scores and tumor mutation burden (TMB), presenting the findings through visualization with the “ggplot2” package in R. Microsatellite instability raw data were extracted from the cbioportal database (http://www.cbioportal.org/). We depicted the mutation landscape and immune infiltration scores in a visual format using the “ComplexHeatmap” R package. This comprehensive analysis aimed to provide insights into the relationship between risk scores, somatic mutations, tumor mutation burden, microsatellite instability, and immune infiltration in colorectal cancer.

### Estimation of the immune microenvironment

The estimation of tumor mesenchymal and immune cell abundances, as well as tumor purity in colorectal cancer, was derived from TCGA expression profiling data using the R software package “Estimate” (Yoshihara et al. 2013). Additionally, data on the composition and abundance of immune cells in the CRC tumor microenvironment were obtained from the TIMER 2.0 database (http://timer.cistrome.org/), which provides results from seven assessment methods. The relative abundance of various immune cell types and immune-related activities in each sample was calculated using single-sample gene set enrichment analysis (ssGSEA). Enrichment analysis was conducted utilizing Metascape (Zhou et al. 2019). Furthermore, 114 metabolic pathways from previous literature were quantified using Gene Set Variation Analysis (GSVA) (Rosario et al. 2018). Metabolic pathways from the KEGG database were also quantified using GSVA. Moreover, the Tumor Stem Cell Index, obtained from a previous study, was utilized to quantify the stemness of tumor samples (Liu et al. 2022a). These comprehensive analyses aimed to provide insights into the tumor microenvironment, immune cell composition, metabolic pathways, and stemness characteristics of colorectal cancer samples.

#### Prediction of immunotherapy and chemotherapy

Xu et al. have curated a web resource containing a collection of genes associated with cancer and the immune cycle (Xu et al. 2018). Additionally, Mariathasan's research has identified a list of genes known for their favorable responses to anti-PD-L1 drugs (Mariathasan et al. 2018). To explore potential correlations between these gene profiles and risk scores, we utilized the GSVA method to quantify both sets of genes. Visualization of these relationships was achieved using the “ggcor” R package. Furthermore, we investigated the relationship between these four model genes and 51 immune-related genes, presenting our findings in a circular heatmap (Thorsson et al. 2019). For the assessment of immune escape likelihood in tumor samples, we employed TIDE (Tumor Immune Dysfunction and Exclusion), a computational framework accessible at http://tide.dfci.harvard.edu/. Immunophenoscores (IPSs) were computed using z-scores for gene expression across four major categories, allowing for the assessment and comparison of potential responses to Immune Checkpoint Inhibitors (ICI) between groups. Higher scores indicate greater immunogenicity. To predict responsiveness to immunotherapy, we examined immunophenotypes (IPS) associated with CRC in The Cancer Immunome Atlas (TCIA) database (https://tcia.at/home) (Charoentong et al. 2017). Moreover, we utilized the “pRRophetic” R software package to determine the half-inhibitory concentration (IC50) of conventional chemotherapeutic drugs for treating colorectal cancer (Geeleher et al. 2014). These comprehensive analyses aimed to provide insights into potential therapeutic responses and immune characteristics associated with CRC, facilitating personalized treatment strategies.

#### Cell culture and knockdown of ACOX1 and CPT2

We acquired the NCM460 normal cell line and CRC cell lines (RKO, HCT116, SW620, and HT29) from the American Type Culture Collection (ATCC). These cells were cultured in RPMI-1640 medium (Gibco, ThermoFisher, USA) supplemented with 10% fetal bovine serum (FBS) (Hyclone, USA) and maintained in a humidified incubator at 37°C with 5% CO2.

To silence the ACOX1 and CPT2 genes, we purchased Small interfering RNAs (siRNAs) from GenePharma (Shanghai, China). Real-time PCR was performed using Taq Pro Universal SYBR qPCR Master Mix (Vazyme, China) on the Applied Biosystems 7300 Real-Time PCR System. The siRNA sequences and primer sequences for ACOX1 and CPT2 are shown in Supplementary Table 2.

For transwell invasion assays, 1×10^5 cells in 500 μl of FBS-free medium were seeded into the upper chamber of 24-well chambers/microfilters coated with Matrigel (BD, Franklin Lakes, NJ, USA). The lower chamber was filled with medium containing 10% FBS for 48 hours. Noninvasive cells on the upper side of the chamber were removed with a cotton swab, and invasive cells were fixed in 4% paraformaldehyde and stained with crystal violet solution. Stained cells were imaged, and the best six fields of view were randomly selected for analysis, with each experiment repeated three times.

Cells from each group were seeded in 96-well culture plates at a density of 3×10^3 cells per well, with three replicate wells for each group. Cell culture supernatants were collected at 0 h, 24 h, 48 h, and 72 h after seeding. Cell viability was assessed using the Cell Counting Kit-8 (CCK-8) assay kit (Sigma-Aldrich, USA) following the manufacturer's instructions. After incubation at 37°C with 5% CO2 for 1 hour, the optical density (OD) at 450 nm was measured using a microplate reader (Bio-Rad, USA), and the cell growth curve was generated.

#### Immunohistochemical validation of experimental findings

The CRC tissue microarray (HCol-Muc060CS-01) used in this study was obtained from Shanghai Xinchao Biotechnology Co. It consisted of 60 CRC tissue samples and their corresponding adjacent non-cancerous tissues from 15 male and 15 female patients with a mean age of 66 ± 12 years. The company's Ethics Committee (Ethics Code: SHYJS-CP-1407013) approved the experimental protocol. Informed consent for the use of tissue samples was obtained from the China Human Genetic Resources Management Office. Our study was conducted in accordance with the Declaration of Helsinki (revised 2013). The experimental protocol consisted of several steps. First, the tissue microarrays were processed for baking, dewaxing, and antigen extraction. Second, ACOX1 and CPT2 primary antibodies (Proteintech Cat# 10957-1-AP/26555-1-AP, 1:2000) were added to the microarrays and incubated at 4 °C overnight. The chip was then contacted with the secondary antibody for 45 minutes at room temperature before staining with DAB and restaining with hematoxylin.

#### Statistical Analysis

All statistical analyses were performed using R version 4.1.3 and Python version 3.9. Prognostic values were computed, and patient survival in distinct subgroups within each dataset was compared through Kaplan-Meier survival analyses, along with log-rank tests. For data that did not adhere to a normal distribution, the Wilcoxon test or Kruskal-Wallis test was applied. For variables demonstrating a normal distribution, two-tailed t-tests or one-way ANOVA were employed to evaluate the clinical characteristics of subgroups with quantitative differences. Spearman correlation analysis was utilized to calculate correlation coefficients. A significance level of *p* < 0.05 was adopted in all statistical analyses.

## Results

### Enrichment score of nucleotide metabolism-related genes in scRNA-seq

**Fig. 1:**
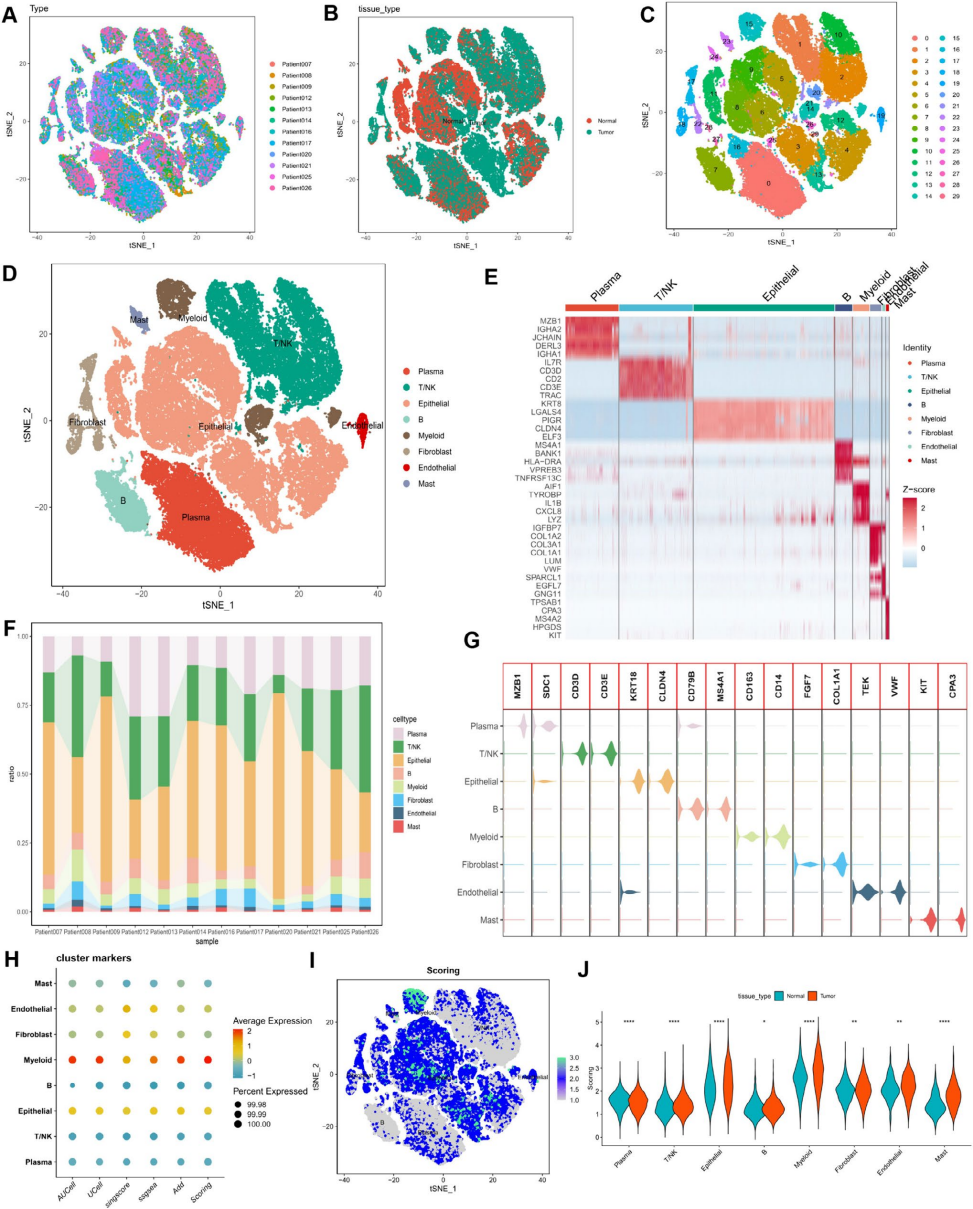
Classification of Cell Subpopulations and Gene Expression Scores Related to Nucleotide Metabolism in Colorectal Cancer. (**A**-**D**) t-SNE plots depicting diverse samples, tissue origins, cell clusters, and cell subpopulations, color-coded for clarity. (**E**) Heatmap illustrating the relative expression of marker genes within eight distinct cell subpopulations. Genes with high expression are represented in red, while those with low expression are displayed in blue. (**F**) Histogram displaying the distribution of cell types across different samples. (**G**) Expression patterns of commonly used marker genes for cellular annotation within these cell subpopulations. (**H**) Bubble plots demonstrating the enrichment scores of nucleotide metabolism-related genes per cell type in colorectal cancer. (**I**) t-SNE plots illustrate the enrichment scores of nucleotide metabolism-related genes for each cell type, with darker shades of green indicating higher scores. (**J**) The discrepancy in enrichment scores of nucleotide metabolism-related genes for each cell type between cancer and normal tissues. ns, Not significant; * *p*< 0.05; ** *p*< 0.01; *** *p*< 0.001; **** *P*< 0.0001

Twelve samples were obtained for the study of colorectal cancer heterogeneity and to assess differences between tumor and normal samples using single-cell sequencing data. Following the identification of 2000 highly variable genes, we employed principal component analysis (PCA) for dimensionality reduction, focusing on the top 20 principal components (PCs). Subsequently, 30 clusters were generated, and known marker genes were referenced to annotate cellular subpopulations. Visualization using t-distributed Stochastic Neighbor Embedding (t-SNE) illustrated distinct samples, tissue types, clusters, and annotated cellular subpopulations (Fig. 1A-D). The relative expression of marker genes, calculated as Top5, in each cellular subpopulation was depicted through a heatmap (Fig. 1E). Furthermore, Fig. 1F displays the percentage distribution of different cell types across the 12 samples. Violin plots were utilized to showcase the expression patterns of common marker genes in each cell type (Fig. 1G). Subsequently, we evaluated the level of nucleotide metabolism in scRNA-seq data based on the expression of 882 nucleotide metabolism-related genes. Using five common algorithms (AddModuleScore, UCell, GSVA, AUCell, and singscore), we scored gene sets to assess nucleotide metabolism. Figs. 1H and 1I demonstrate that nucleotide metabolism scores (NMS) were relatively higher in epithelial and myeloid cells. Additionally, we compared NMS across various cell types in tumor and normal samples, revealing that NMS in cells such as myeloid cells, fibroblasts, T/NK cells, and epithelial cells was relatively higher in tumors (Fig. 1J). Furthermore, a differential analysis was conducted for epithelial cells in the tumor and normal tissues, identifying a total of 102 NMRGs with expression differences in both settings (Supplementary Table 3).

#### Analysis of cellular interactions in scRNA-seq

**Fig. 2:**
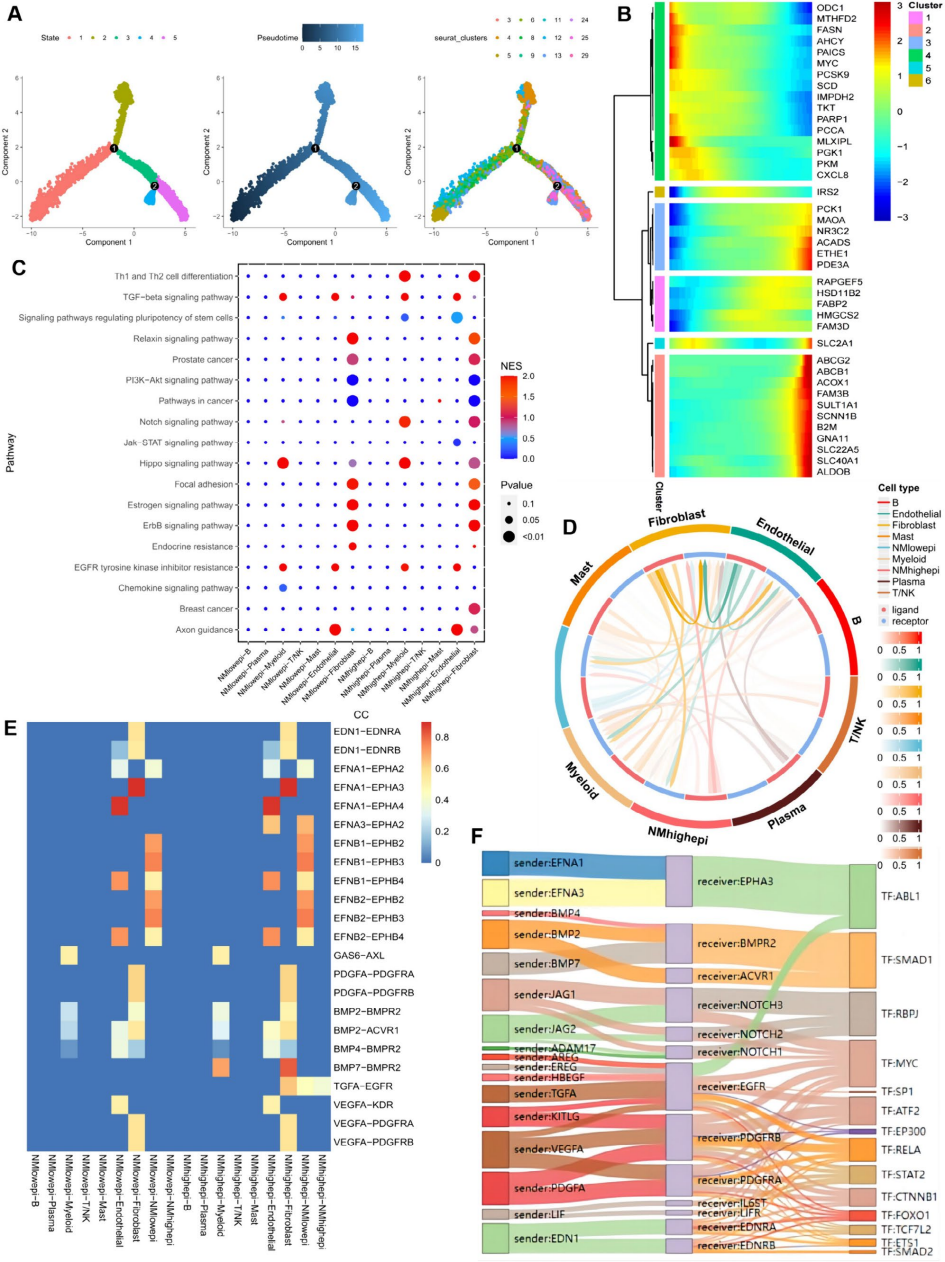
Cell Developmental Trajectory Analysis and Cell Communication Analysis. (**A**) Cell trajectory and pseudo-time analysis for malignant cells. (**B**) Heatmap illustrating the expression patterns of 40 genes related to nucleotide metabolism that exhibit differential expression during cell development. Low expression is represented in blue, while high expression is depicted in red. (**C**) Bubble diagram showcasing the activity analysis of signaling pathways across various cell types. (**D**) Circle diagram visualizing the strength of ligand-receptor interactions between different cell types. (**E**) Identification of highly ranked ligand-receptor pairs and their associated transcription factors between epithelial cells and fibroblasts. (**F**) Assessment of ligand-receptor strength between diverse cell types

Cell trajectory analysis provides valuable insights into cellular differentiation relationships, developmental trajectories, and changes in tumor immune cell dynamics at single-cell resolution. In our study, we utilized the “monocle” R package to determine cell trajectories and pseudotime distributions of epithelial cells in tumor tissues, identifying a total of five cell states in epithelial cells during development, with cluster 5 corresponding to the end state of cell development (Fig. 2A). The heatmap in Fig. 2B illustrates the expression patterns of the top 40 nucleotide metabolism-related genes with the highest differential expression at various stages of epithelial cell development, highlighting, for instance, the predominantly high expression of ACOX1 at the end of epithelial cell development. We classified epithelial cells into nucleotide metabolism (NM) high and NM low groups based on the median value of Nucleotide Metabolism Score (NMS) in all epithelial cells in tumor tissues. Bubble plots were then utilized to visualize the results of signaling pathway activity analysis (Fig. 2C), revealing, for instance, enhanced Notch signaling pathway activity in epithelial cells with high nucleotide metabolism scores, particularly with fibroblasts. From a molecular pathology perspective, the accumulation of DNA mutations, particularly in molecules within the Notch signaling pathway, plays a crucial role in malignancy development (Meurette and Mehlen 2018). The circled graph in Fig. 2D demonstrates the strength of ligand-receptor signaling between different cell types, with epithelial cells exhibiting higher nucleotide metabolism scores showing stronger cellular communication with fibroblasts. Further analysis revealed intensified ligand-receptor pair relationships between high-scoring epithelial cells and fibroblasts, exemplified by the TGFA-EGFR interaction (Fig. 2E). The abnormal activation of the TGF-α/EGFR autocrine loop, observed in many malignant tumors, underscores its close association with tumorigenesis and progression (Tang et al. 2016). Finally, we inferred the existence of ligand-receptor relationships and corresponding transcription factors (TFs) between epithelial cells and fibroblasts with higher nucleotide metabolism scores (Fig. 2F), shedding light on potential regulatory mechanisms underlying cellular interactions in the tumor microenvironment

#### Characterization of nucleotide metabolism in spatial transcriptome sequencing

**Fig. 3:**
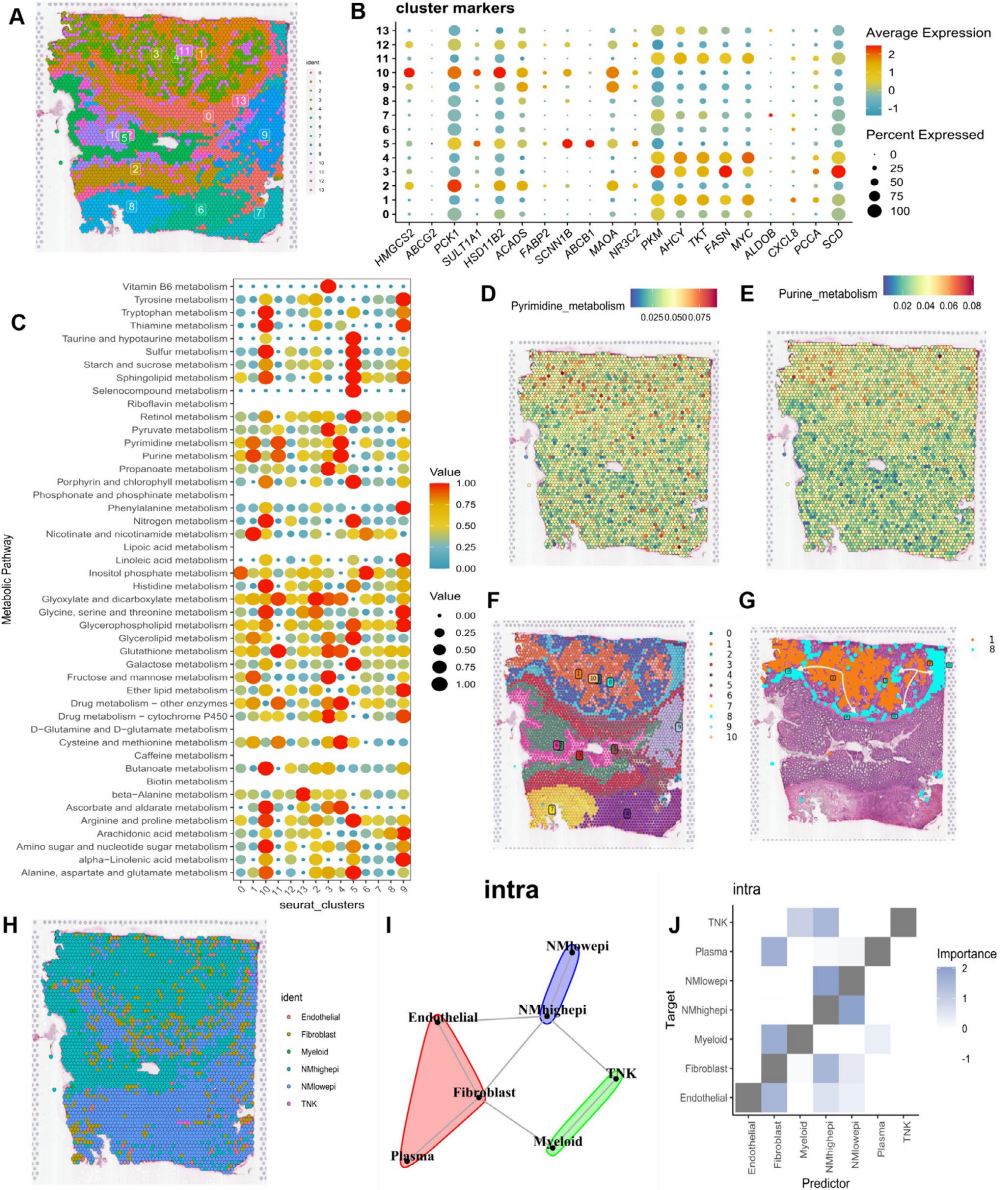
Characterization of nucleotide metabolism in the spatial transcriptome of CRC. (**A**) Spatial representation illustrating the identification of 14 clusters through stRNA-seq. (**B**) Bubble plot displaying the expression levels of genes related to nucleotide metabolism within distinct clusters. Red signifies high expression, while blue indicates low expression. (**C**) Bubble chart presenting the metabolic intensity across various clusters. (**D**) Spatial depiction of pyrimidine metabolism intensity. (**E**) Spatial visualization of purine metabolic intensity. (**F**) Spatial representation of the 11 cellular clusters identified using Python. (**G**) Spatial map showcasing the developmental trajectory of clusters 1 through 8. (**H**) An algorithm is used to identify the predominant distribution of different cell types within the CRC spatial map using RCTD. (**I**) Extrapolation of spatial clustering of different cell types based on MISTy. (**J**) Projection of spatial correlations among different cell types based on MISTy

We employed SCTransform's approach to correct for spatial sequencing depth and conducted a series of normalization processes, resulting in the identification of 14 distinct cellular subpopulations in space following dimensionality reduction clustering (Fig. 3A). Notably, subpopulations 1, 3, 4, and 11 were predominantly situated in the tumor core of colorectal cancer, as depicted in the original representation of the spatial transcriptome. Bubble plots showcasing the expression patterns of the top 20 nucleotide metabolism-related genes with the largest expression differences are illustrated in Fig. 3B. The metabolic activity of different cell subpopulations was further analyzed using the “scMetabolism” R package. Subpopulations 1, 3, 4, and 11, located in the core region of the tumor, exhibited close associations with purine and pyrimidine metabolic activities (Fig. 3C). This metabolic activity enrichment was primarily observed in the core region of the tumor, as depicted in Figs. 3D and 3E. Subsequently, employing Python's Scanpy and stlearn packages, we conducted cell developmental trajectory analysis on spatially resolved cell subpopulations. Following normalization and clustering of spatial transcriptome data, a total of 11 distinct cell subpopulations were identified (Fig. 3F). Intriguingly, cluster 1, situated in the core region of the tumor, exhibited differentiation towards cluster 8 in the peripheral region of the tumor, as observed in the trajectory analysis (Fig. 3G). Furthermore, we employed the RCTD method to back-convolute annotated cell types from single-cell data to spatial data, inferring the predominant cell types at each spatial location. Epithelial cells with high nucleotide metabolism scores were primarily located in the core region of the tumor, whereas those with low scores were predominantly concentrated in the peripheral region of the tumor (Fig. 3H). Finally, according to MISTy's results, epithelial cells with higher nucleotide metabolism scores showed congruence of clustering and higher correlation of spatial interactions with fibroblasts in the internal space (Fig. 3I, J).

### Construction of a prognostic model related to nucleotide metabolism

**Fig. 4:**
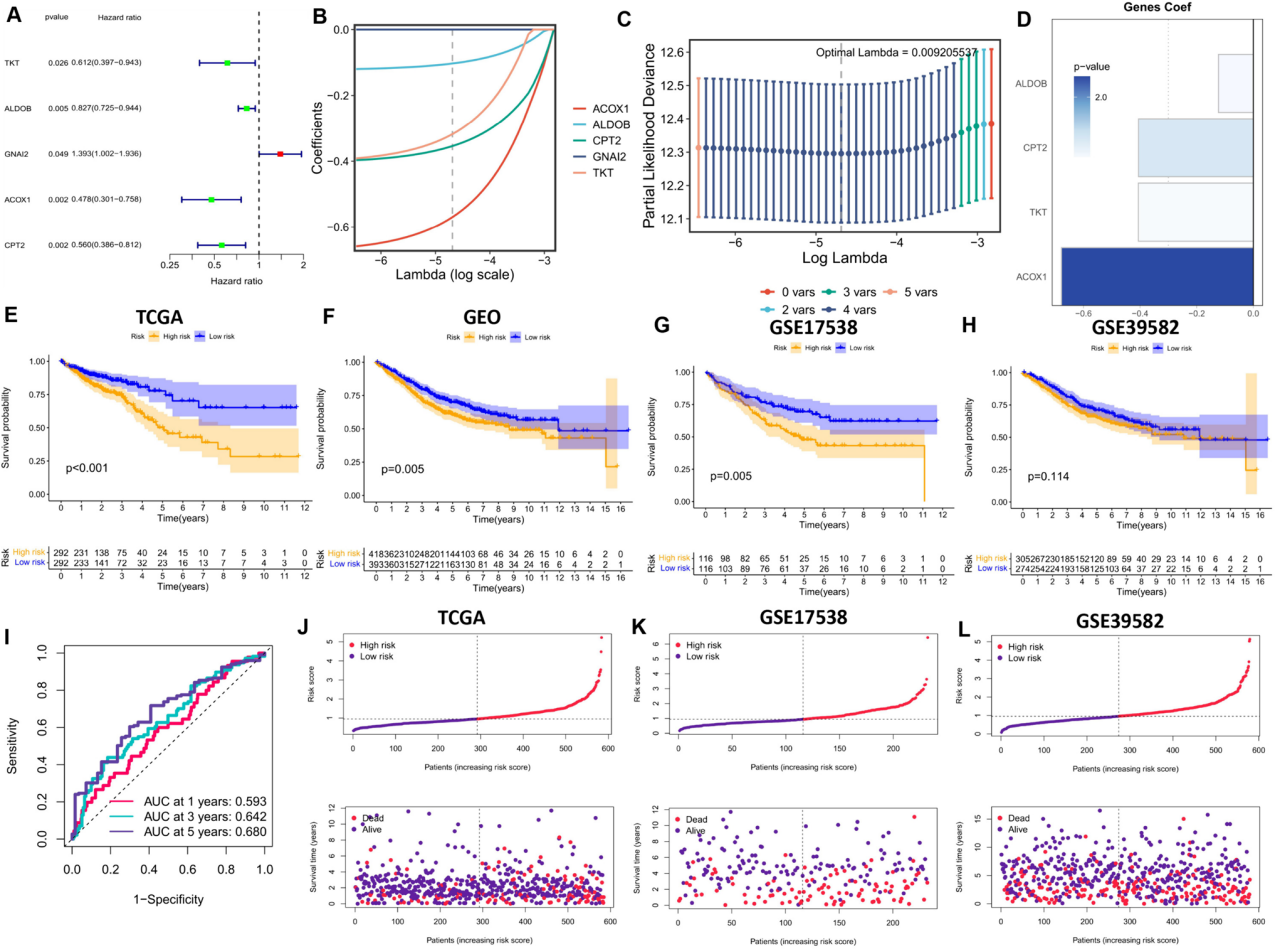
Calculation of Risk Scores Associated with Nucleotide Metabolism and Development of Prognostic Models. (**A**) Forest plot presenting the five prognostic genes identified through univariate Cox analysis. (**B**) Profiles of LASSO coefficients. (**C**) Ten-fold cross-validation for selecting tuning parameters in the LASSO model. (**D**) Results of multivariate Cox analysis for model genes and their corresponding coefficients. (**E**-**H**) Kaplan-Meier survival curves for overall survival (OS) of patients categorized into low-risk and high-risk groups in the TCGA cohort, the complete GEO cohort, the GSE17538 cohort, and the GSE39582 cohort. (**I**) Area under the curve (AUC) values for risk scores at 1, 3, and 5 years in the TCGA cohort. (**J**-**L**) Distribution of scores among low-risk and high-risk groups in the TCGA cohort, the GSE17538 cohort, and the GSE39582 cohort, along with patient survival data

To leverage the potential of nucleotide metabolism-related gene signatures for clinical decision support, we utilized 102 expression-differentiated NMRGs to develop prognostic models for colorectal cancer. These models were constructed using both high-throughput sequencing data and microarray data. Our methodology involved utilizing a training set consisting of 584 CRC samples with available survival data from the TCGA dataset to construct prognostic risk models. Additionally, we employed 232 and 579 CRC patient samples with survival information from the GSE17538 and GSE39582 cohorts for external validation. Initially, we conducted a Univariate Cox analysis to identify five NMRGs significantly influencing OS in CRC patients (Fig. 4A). To address the risk of overfitting and refine the gene selection for OS prediction, LASSO regression analysis was performed, resulting in the selection of four candidate genes from the initial five (Fig. 4B and C). Subsequently, stepwise multifactorial Cox analysis identified ACOX1, ALDOB, CPT2, and TKT as independent prognostic factors. The risk score was then calculated by summing the expression levels of individual genes, each weighted by their corresponding regression coefficients (Fig. 4D). Patients were stratified into low-risk and high-risk groups using the median score as the cutoff point. Survival analyses conducted for both the TCGA-trained group and the GEO-validated group consistently demonstrated that patients in the high-risk category exhibited poorer OS compared to those in the low-risk group (Fig. 4E-H). Additionally, high-risk patients displayed worse progression-free survival (PFS) (Supplementary Figure 1A). Furthermore, receiver operating characteristic curves illustrated the strong predictive capability of the risk score for OS in the TCGA cohort, as depicted in Fig. 4I. The risk plots provided detailed survival outcomes for individual patients across the TCGA cohort, as well as the GSE17538 and GSE39582 cohorts (Fig. 4J-L). These findings underscore the potential clinical utility of our prognostic models based on nucleotide metabolism-related gene signatures, offering valuable insights for personalized treatment strategies in CRC patients.

### Validation of clinical features and construction of nomograms

**Fig. 5:**
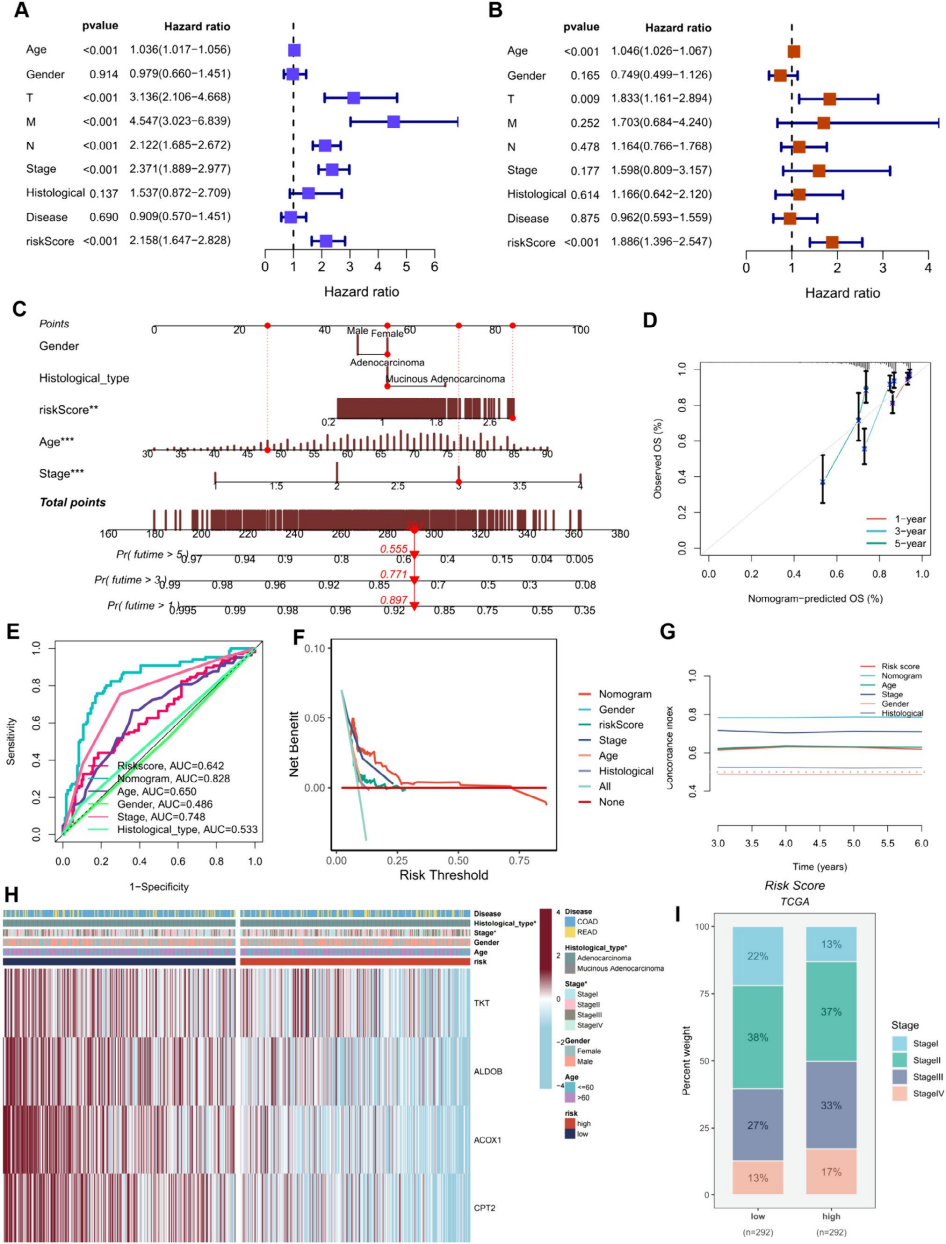
Independent Prognostic Analysis of Risk Scores and Clinicopathologic Factors in the TCGA Cohort. (**A**, **B**) Univariate and multivariate Cox regression analyses of clinicopathologic variables and risk scores for overall survival (OS) in the TCGA training cohort. (**C**) Integrated nomograms combining age, grade, and stage for the prediction of OS at 1, 3, and 5 years in colorectal cancer patients. (**D**) Calibration curves for the nomograms. (**E**) Area under the curve (AUC) values for risk scores and clinical characteristics at 3 years in the TCGA cohort. (**F**) Decision curve analysis (DCA) curves for risk scores, nomogram scores, and other clinical characteristics. (**G**) Assessment of predictive performance using C-Index for different clinical characteristics, nomogram scores, and risk scores. (**H**) Heatmap displaying the expression profiles of the four model genes and clinical characteristics associated with subgroups, as determined by the chi-square test. (**I**) Distribution of clinical stages within various score subgroups. ns, Not significant; * *p*< 0.05; ** *p*< 0.01; *** *p*< 0.001; **** *P*< 0.0001

Considering the robust correlation observed between our nucleotide metabolism-based risk model and adverse prognosis, we sought to assess the potential of our 4-NMRG signature as an independent prognostic predictor in colorectal cancer patients. In the TCGA cohort, we conducted univariate Cox analysis, revealing that the risk score could serve as an independent prognostic indicator, surpassing other common clinical characteristics (such as age, grade, stage, and histologic type) (Fig. 5A). This trend persisted even after multifactorial analysis, further establishing the risk score as the most reliable independent predictor within the cohort (Fig. 5B). Consistent with these findings, in the GEO external validation cohort, the risk score demonstrated its potential as an independent prognostic indicator for patients (Supplementary Figure 1B, C). To enhance the clinical utility of our risk model and aid clinicians in making informed decisions, we developed a nomogram for predicting 1-, 3-, and 5-year survival rates for CRC patients based on correlations between clinicopathologic features and risk scores (Fig. 5C). The risk score displayed a greater impact on OS prediction, underscoring the superior prognostic potential of our 4-NMRG-based risk model. Calibration curves affirmed the accuracy of the nomogram’s predictions (Fig. 5D), while the area under the curve (AUC) at 3 years significantly outperformed other clinicopathologic features (Fig. 5E). Decisions Curve Analysis curves at 3 years (Fig. 5F) and C-index values (Fig. 5G) consistently demonstrated that our constructed nomograms and risk scores provided the highest net benefit, surpassing traditional models, thus wielding more substantial influence on clinical decision-making. We further illustrated associations between risk groupings, clinical characteristics, and the expression of our four modeled genes using heatmaps. Chi-square tests revealed significant associations between risk groupings and patient Stage and histologic type. Interestingly, all four model genes exhibited higher expression in the low-risk group (Fig. 5H), and the high-risk group displayed more advanced Stage staging (Fig. 5I). These analyses reinforce the reliability of the risk score and nomogram as a clinical predictive scoring system.

### Mutational landscape and microsatellite instability

**Fig. 6:**
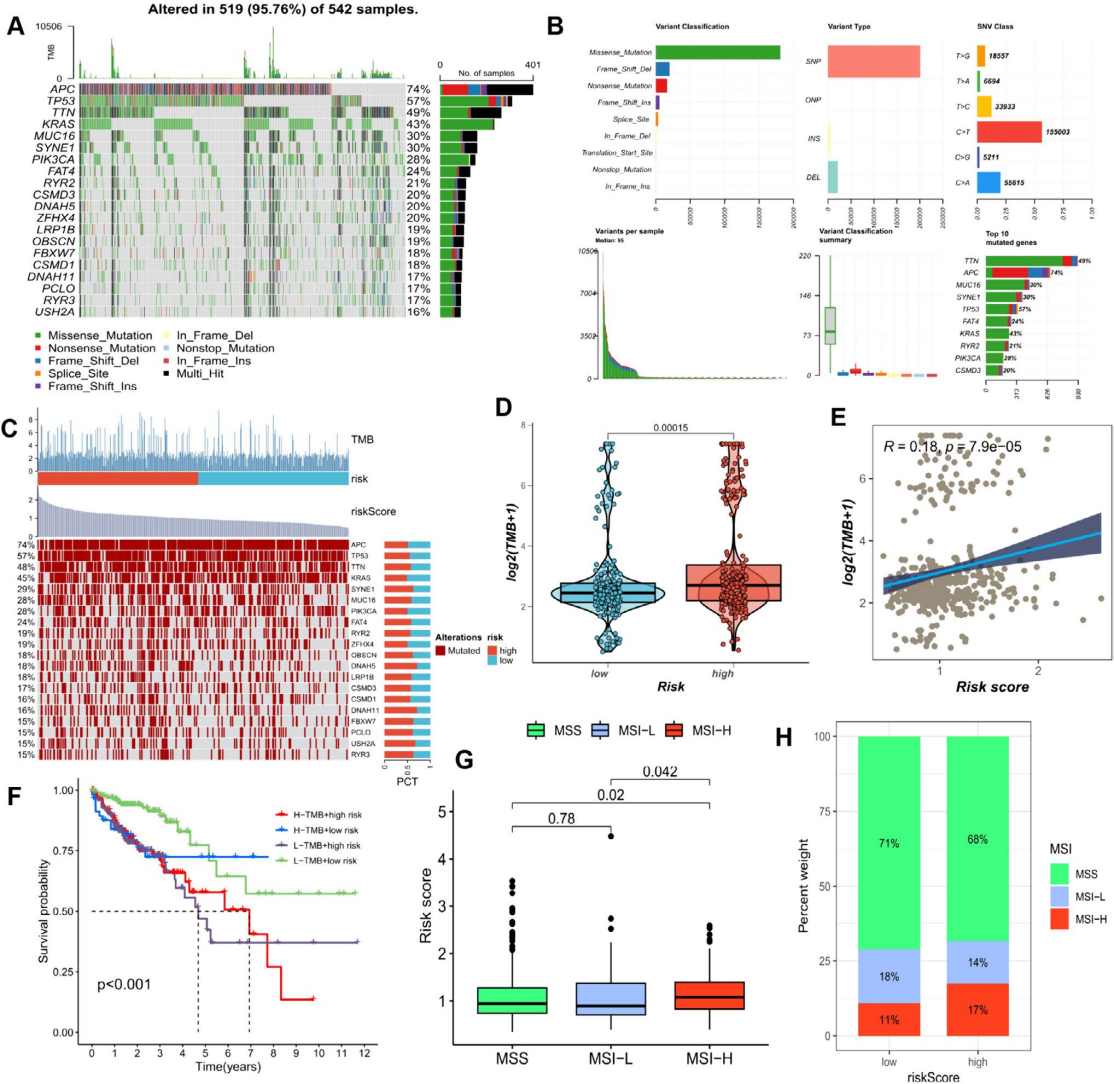
Mutational Landscape and Microsatellite Instability in CRC Samples. (**A**) Overview of the mutation landscape in 542 CRC samples. (**B**) Detailed breakdown of mutation types, with missense mutations being the most common. Single-nucleotide polymorphisms (SNPs) constituted the majority of mutations, with C>T mutations occurring most frequently. Horizontal histograms present the top 10 mutated genes in CRC. (**C**) Mutation status and tumor mutation load (TMB) of the 20 genes with the highest mutation frequency across different risk subgroups. (**D**) Comparison of TMB among different subgroups. (**E**) Correlation analysis between risk scores and TMB. (**F**) Survival disparities among four subgroups: H-TMB+ high-risk score, H-TMB+ low-risk score, L-TMB+ high-risk score, and L-TMB+ low-risk score. (**G**) Differences in risk scores of CRC patients in three subgroups based on microsatellite instability: microsatellite high instability (MSI-H), microsatellite low instability (MSI-L), and microsatellite stable (MSS). (**H**) Percentage of MSI classifications for patients in high-risk and low-risk groups

The efficacy of immunotherapy often varies greatly among patients with different tumors, and in addition to factors related to tumor type, pathological stage, and immune infiltration, genetic mutations may also affect the efficacy of immunotherapy. The waterfall plot in Fig. 6A illustrates the somatic mutation spectrum of CRC patients, where the most frequent form of mutation in these genes is missense mutation. Figure 6B includes statistical plots of various mutation classifications, summary plots of base alterations, box plots of the various mutation classifications in the samples, statistical plots of the 10 genes with the highest number of mutations, plots of the number of mutation counts included in each sample, and the sample percentage profile. We examined the distribution of the most commonly mutated genes in CRC in risk score subgroups (Fig. 6C). Patients in the high-risk score subgroup exhibited higher tumor mutation load (TMB) relative to patients in the low-risk score subgroup (Fig. 6D, E). Next, we categorized patients into four groups based on median TMB values and median risk scores (high-TMB+ high-risk score, high-TMB+ low-risk score, low-TMB+ high-risk score, and low-TMB+ low-risk score), and the results showed that patients with high-risk scores and low mutations had the relatively worst OS (Fig. 6F). When the DNA mismatch repair function is aberrant, replication errors occurring in the microsatellites result in microsatellite instability (MSI). Microsatellite instability is categorized into Microsatellite High Instability (MSI-H), Microsatellite Low Instability (MSI-L), and Microsatellite Stable (MSS) based on degree. Compared to the MSI-low group, the MSI-high group in CRC had a greater risk score (Fig. 6G, H).

### Prediction of immune infiltration and biological mechanisms

The tumor microenvironment (TME) is a critical determinant of patient clinical outcomes and therapeutic response. Tumor-infiltrating lymphocytes (TILs), comprising various cell types such as effector, regulatory, and inflammatory cells, engage in complex interactions mediated by cytokines and soluble factors. Additionally, tumor cells themselves release immunosuppressive cytokines, influencing immune cell recruitment within the microenvironment. Consequently, the composition of cells and their interactions with cytokines in the TME collectively shape the anti-tumor immune response (Xie et al. 2022).

**Fig. 7:**
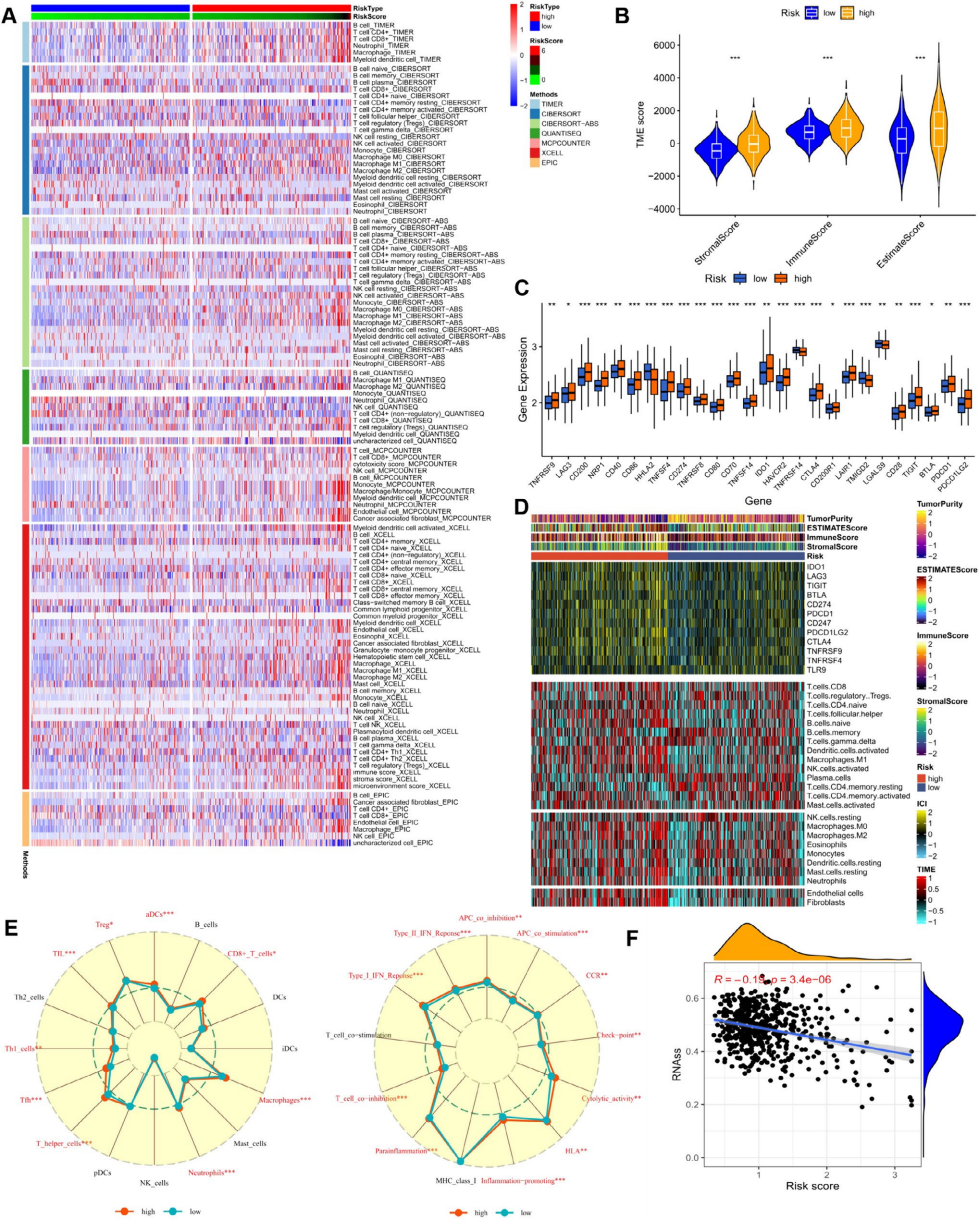
Analysis of the immune microenvironment and immune-related functions in different risk-scoring subgroups of the TCGA cohort. (**A**) Evaluation of variations in immune infiltration across risk score subgroups employing seven different algorithms. (**B**) Assessment of differences in immune scores and stromal scores calculated via ESTIMATE for distinct risk score subgroups. (**C**) Examination of variations in immune checkpoint expression within different risk score subgroups. (**D**) Heatmap displaying distinctions in tumor microenvironment (TME) score, immune checkpoint expression, and immune cell infiltration among diverse risk subgroups. (**E**) Radar chart depicting variations in immune cell infiltration and immune-related pathways assessed via ssGSEA among patients in different risk groups. (**F**) Correlation analysis between cancer RNA stemness score (RNAss) and risk score. ns, Not significant; * *p*< 0.05; ** *p*< 0.01; *** *p*< 0.001; **** *P*< 0.0001

In this study, we explored the immune landscape of both high and low-risk score groups using various algorithms (Fig. 7A). To delve deeper into the relationship between risk scores and immune-related functions, we evaluated the enrichment scores of different immune cell subpopulations and functions using the ssGSEA approach. Our findings revealed that the high-risk score group exhibited heightened infiltration scores of immune cells and elevated immune pathway scores (Fig. 7E). We employed the “estimate” method to estimate tumor purity by calculating stromal and immune cell ratios across different risk groups (Fig. 7B). Considering the significant impact of immune checkpoint molecules on tumor immunotherapy, we analyzed the expression of immune checkpoint genes within distinct risk score subgroups, revealing higher expression levels across almost all immune checkpoints in the high-risk score group (Fig. 7C). A heatmap depicted immune checkpoint genes, immune scores, immune cell infiltration, and tumor microenvironment scores across various risk score groups (Fig. 7D).

Furthermore, we investigated the correlation between the RNA stemness score (RNAss) and the risk score, uncovering a notable negative correlation (Fig. 7F). This suggests that CRC cells with lower risk scores exhibit more prominent stem cell characteristics and lower levels of cell differentiation. These findings imply that patients in the high-risk score group may have a less favorable prognosis, accompanied by heightened immune activity, likely indicative of an immunosuppressive tumor microenvironment in colorectal cancer. This could potentially result in a reduced response rate to immunotherapy.

**Fig. 8:**
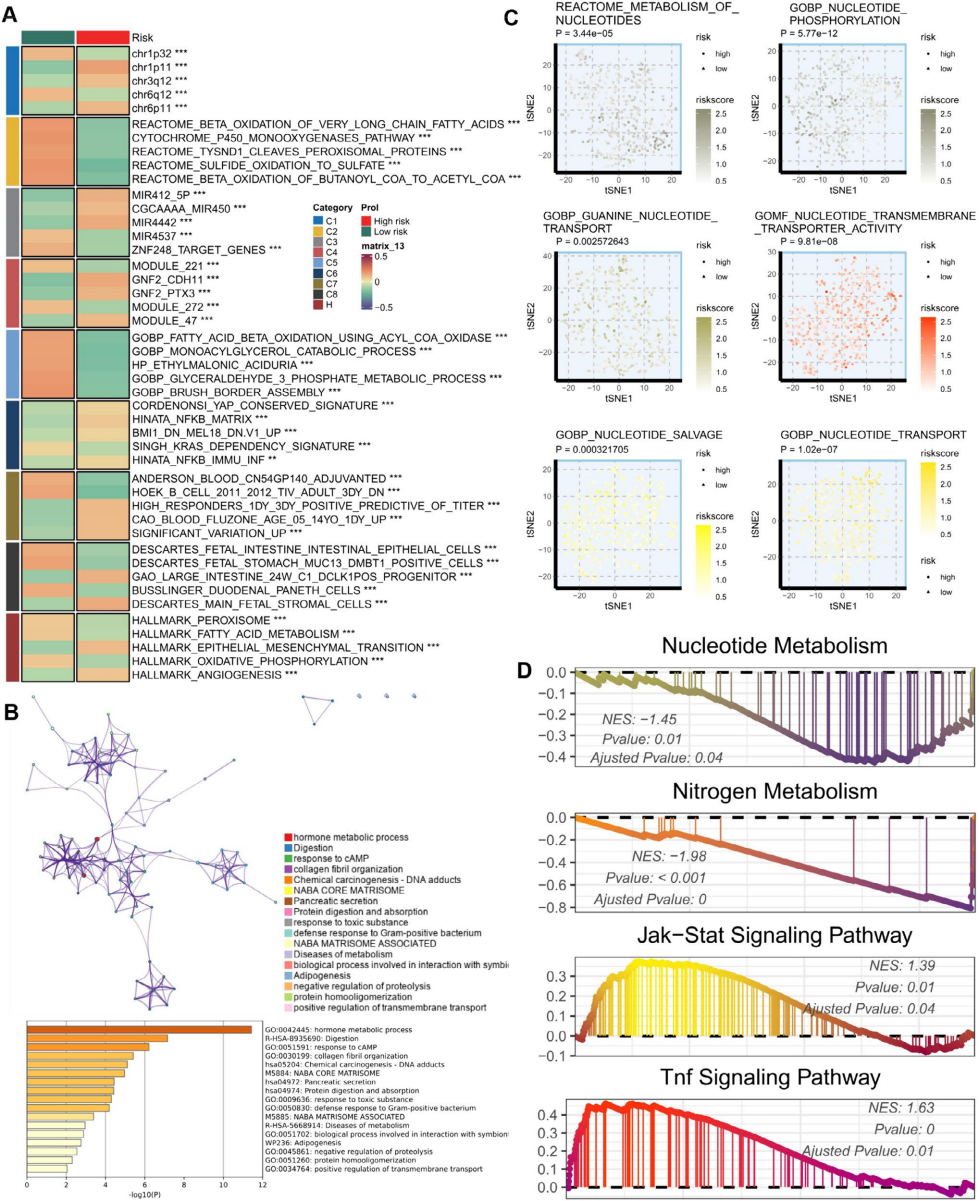
Biological characteristics of different risk score groups in the TCGA cohort. (**A**) MsigDB-based GSVA analysis describing the biological properties of the two nucleotide metabolism-related score groups. (**B**) Metascape-based enrichment analysis of differentially expressed genes between the two risk-scoring groups. (**C**) t-SNE plots of both GO and Reactome terms describing the differences in nucleotide metabolic pathway activities in the two risk-scoring groups. (**D**) . GSEA of GO and KEGG terms for the risk signature. ns, Not significant; * *p*< 0.05; ** *p*< 0.01; *** *p*< 0.001; **** *P*< 0.0001

Moreover, our risk score signature demonstrated a strong positive correlation with various tumorigenic pathways, including epithelial-mesenchymal transition, angiogenesis, and NF-KB signaling pathways (Fig. 8A). Notably, we observed significant distinctions in nucleotide metabolism-related pathways between the risk groups (Fig. 8B). Differentially expressed genes (DEGs) between the two nucleotide metabolism-related risk subgroups were enriched in hormone metabolism and metabolism-related diseases (Fig. 8C). In terms of the Gene Ontology (GO) and Kyoto Encyclopedia of Genes and Genomes (KEGG) terms derived from Gene Set Enrichment Analysis (GSEA), we noted distinct enrichment patterns between the low-risk and high-risk groups. Specifically, the low-risk group exhibited enrichment in nucleotide metabolism and nitrogen metabolism, while the high-risk group showed significant enrichment in JAK-STAT and tumor necrosis factor (TNF) signaling pathways (Fig. 8D).

### Prediction of the effects of immunotherapy and chemotherapy

Tumor immunotherapy, particularly immune checkpoint inhibitors (ICBs), has transformed cancer treatment by activating T-cells, reversing CD8 T-cell depletion, and stimulating immune cells to recognize and eliminate tumor cells. However, the effectiveness of ICB is limited to a subset of tumor patients, with many not experiencing long-term benefits (Xiong et al. 2023). To deepen our understanding of how risk scores influence immunotherapy, we utilized TIDE and IPS scores to evaluate patients with tumors and regional lymph nodes, assessing their potential for an immunocompetent response. This approach aimed to more effectively identify suitable candidates for immunotherapy, recognizing that the efficacy of immunotherapy can vary depending on the level of immune infiltration, often influenced by tumor progression. Expanding on these findings, we explored the feasibility of a prognostic model for predicting the response to immune checkpoint blockade (ICB) in colorectal cancer patients.

**Fig. 9:**
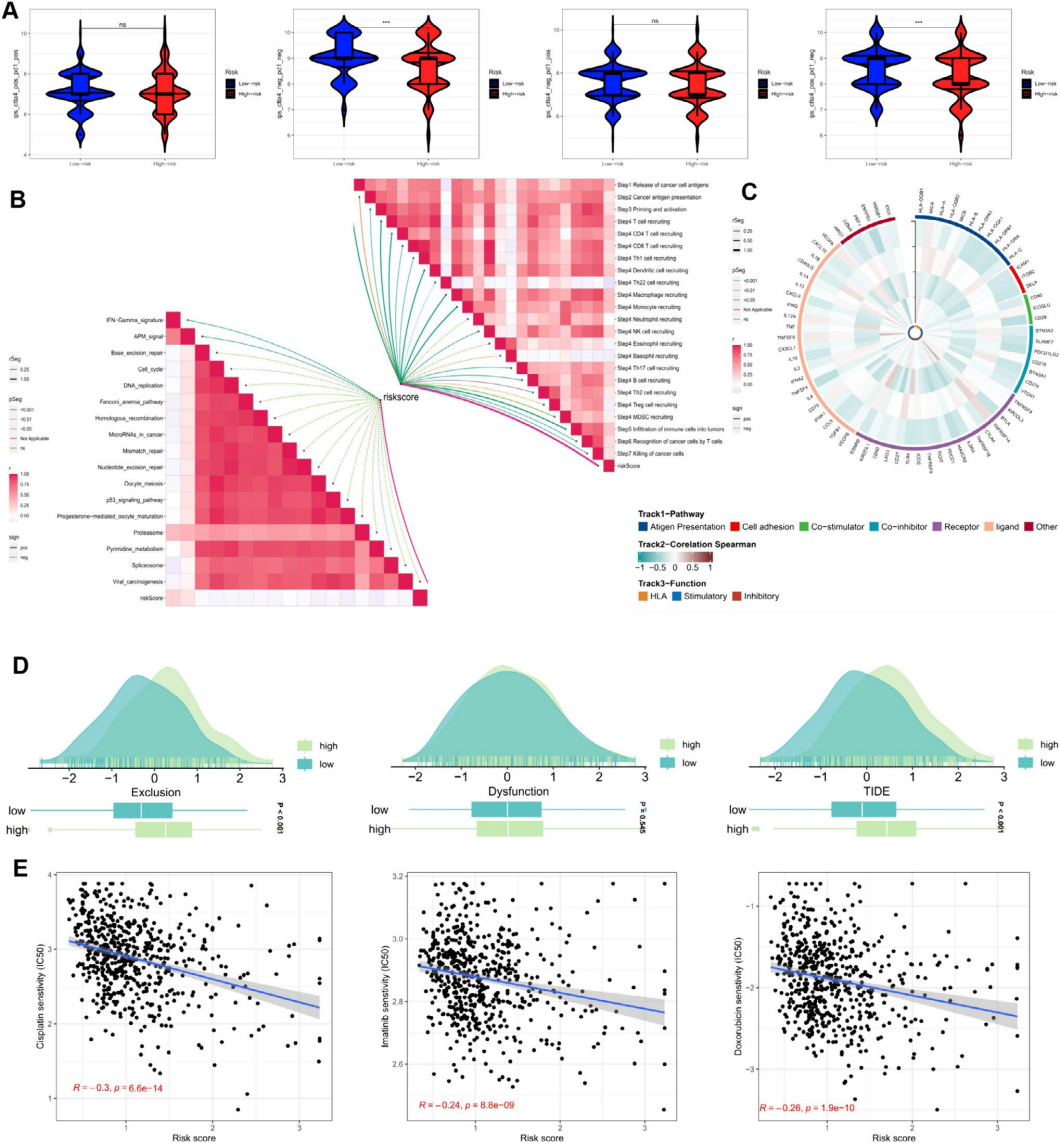
Prediction of the effects of immunotherapy and chemotherapy. (**A**) Comparison of the relative distributions of immunization scores (IPS) in the high-risk scoring group and the low-risk scoring group. (**B**) The relationship between risk scores, ICB response traits, and the various stages of the tumor-immunity cycle. (**C**) Heatmap of modeled gene-immunity gene correlations. (**D**) Differences in TIDE between CRC patients in the high-risk scoring group and those in the low-risk scoring group. (**E**) Correlation of risk scores with IC50 values for cisplatin, imatinib, and doxorubicin. ns, Not significant; * *p*< 0.05; ** *p*< 0.01; *** *p*< 0.001; **** *P*< 0.0001

The violin plot in Fig. 9A illustrates the relationship between IPS and risk groups, with higher IPS values indicating an improved likelihood of responsiveness to PD-1 and CTLA-4 inhibitors. Notably, individuals in the low-risk group exhibited superior immune responses to immune checkpoint inhibitors, particularly CTLA-4 inhibitors. Since the immune microenvironment modulates ICB responses, we conducted an in-depth analysis of the correlation between risk scores and ICB response characteristics. Our findings revealed a significant positive correlation between the risk score and proteasome and APM_signal while displaying a significant negative correlation with other ICB response attributes. Furthermore, the risk score exhibited substantial and meaningful associations with key stages of the tumor immune cycle, including cancer cell antigen release (step 1), cancer antigen presentation (step 2), initiation and activation (step 3), and immune cell infiltration into the tumor (step 4) (Fig. 9B).

To further investigate variations in immune responses among different subgroups, we conducted correlation analyses involving 12 model genes and classical immune-related genes (Fig. 9C). Higher tumor TIDE prediction scores are associated with reduced responsiveness to immune checkpoint blockade (ICB) and diminished patient survival. Our findings revealed that individuals in the high-risk score group demonstrated elevated dysfunction and exclusion scores, along with relatively higher TIDE scores, as depicted in Fig. 9D. Lastly, we explored the relationship between risk scores and the IC50 values of three clinically utilized chemotherapeutic agents. Our findings showed a significant negative correlation between risk scores and the IC50 values of cisplatin, imatinib, and doxorubicin (Fig. 9E). Taken together, these results suggest that individuals in the low-risk group may be more likely to benefit from both immunotherapy and chemotherapy.

### ACOX1+ and CPT2+ tumor cells may serve as prognostic influencers and targets for immunotherapy

**Fig. 10:**
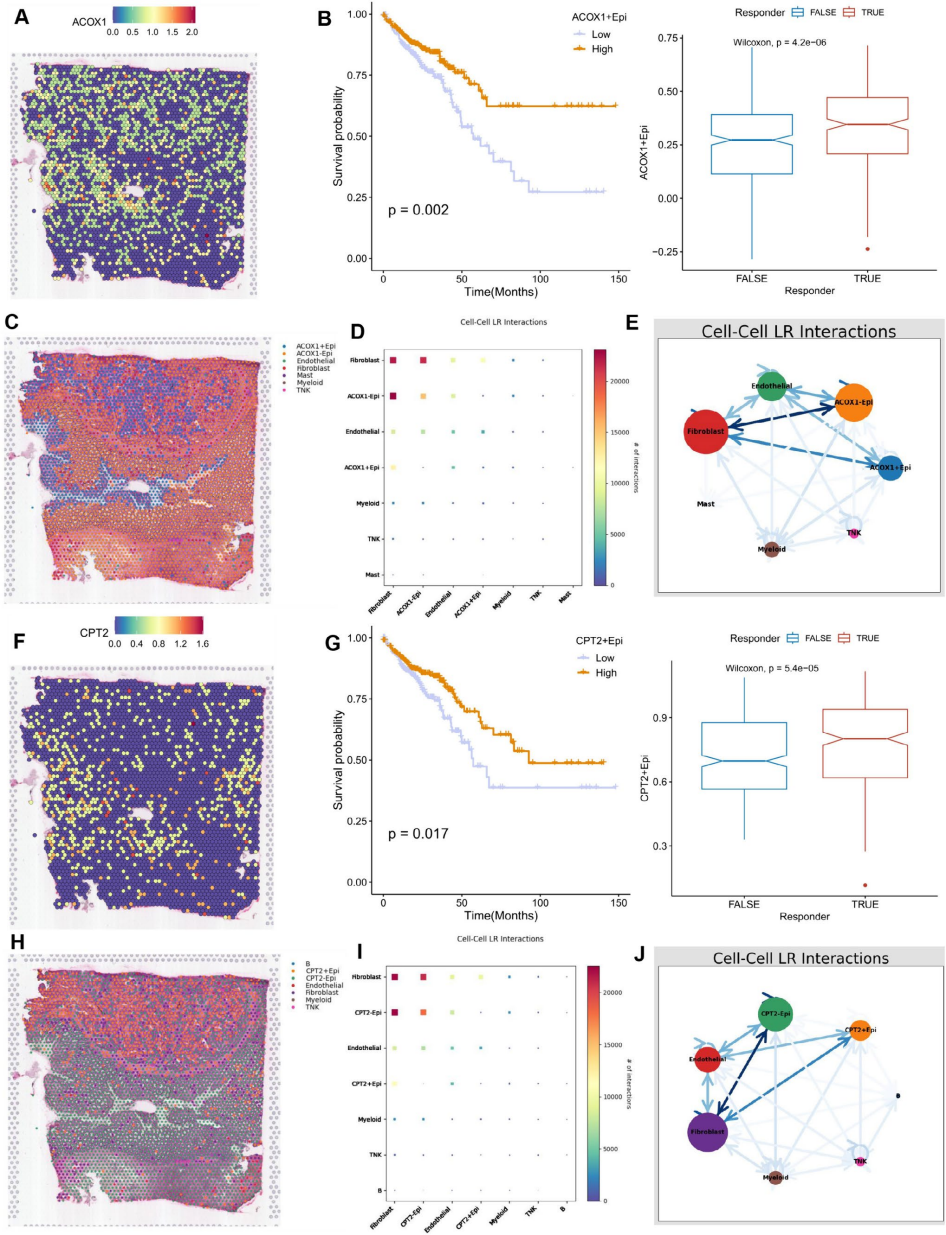
ACOX1 and CPT2 are protective genes in CRC patients. (**A**) Spatial map demonstrating the expression of ACOX1 in colorectal cancer. (**B**) Kaplan-Meier survival curves of OS for patients in the ACOX1+ epithelial cells high and low expression groups. The proportion of ACOX1+ epithelial cells in patients producing different immune responses. (**C**) Spatial maps of different cell types were obtained by the algorithm of reverse convolution. Included here are ACOX1 expression-positive and expression-negative epithelial cells, endothelial cells, myeloid cells, mast cells, fibroblasts, and T/NK cells. (**D**, **E**) Heatmaps and network diagrams to predict the strength of communication between different cell types based on the stlearn method. (**F**) Spatial map demonstrating the expression of CPT2 in colorectal cancer. (**G**) Kaplan-Meier survival curves for OS in patients in the CPT2+ epithelial cells high and low expression groups. The proportion of CPT2+ epithelial cells in patients who produced different immune responses. (**H**) Spatial maps of different cell types were obtained by an algorithm of reverse convolution. Included here are CPT2 expressing positive and expressing negative epithelial cells, fibroblasts, T/NK cells, endothelial cells, myeloid cells, and B cells. (**I**, **J**) Heatmaps and network diagrams of the strength of communication between different cell types were extrapolated according to the method of stlearn

A total of four nucleotide metabolism-related genes (ACOX1, ALDOB, CPT2, and TKT) were incorporated into our risk model. The spatial maps illustrate the expression patterns of these four genes (Fig. 10A, F, Supplementary Figure 2A, C). In our scRNA-seq analysis, we categorized epithelial cells into two groups: those expressing the four genes (expression-positive) and those not expressing them (expression-negative). Utilizing the ssGSEA algorithm, we estimated the abundance of these cells in the TCGA dataset based on marker genes specific to the positively expressing epithelial cells. For survival analysis, we determined the optimal cutoff value and subsequently divided the CRC patients from the TCGA dataset into two groups.

Patients with higher proportions of ACOX1+ and CPT2+ epithelial cells exhibited relatively more favorable prognosis and a higher likelihood of responding effectively to immunotherapy (Fig. 10B, G). Conversely, patients with higher proportions of TKT+ epithelial cells had a relatively better prognosis. However, the difference in cell proportions between patients who did or did not produce an effective immune response, as indicated by TIDE analysis, was not statistically significant (Supplementary Figure 2B). Although patients with a higher proportion of ALDOB+ epithelial cells also demonstrated a relatively better overall survival, the log-rank test results showed no significant difference between the two groups (Supplementary Figure 2D). These findings suggest that ACOX1 and CPT2 expression-positive epithelial cells might serve as protective factors for colorectal cancer patients.

The Reverse Cell Type Delineation (RCTD) method was employed to back-convolute well-annotated cell types from the single-cell data into spatial data (Fig. 10C, H). Interestingly, the extrapolation of cell communication relationships in space revealed that ACOX1-negative expressing epithelial cells exhibited stronger cellular interactions with fibroblasts compared to ACOX1-expressing positive epithelial cells (Fig. 10D, E). Similarly, CPT2-negative expressing epithelial cells also displayed enhanced cellular communication with fibroblasts (Fig. 10I, J).

**Fig. 11:**
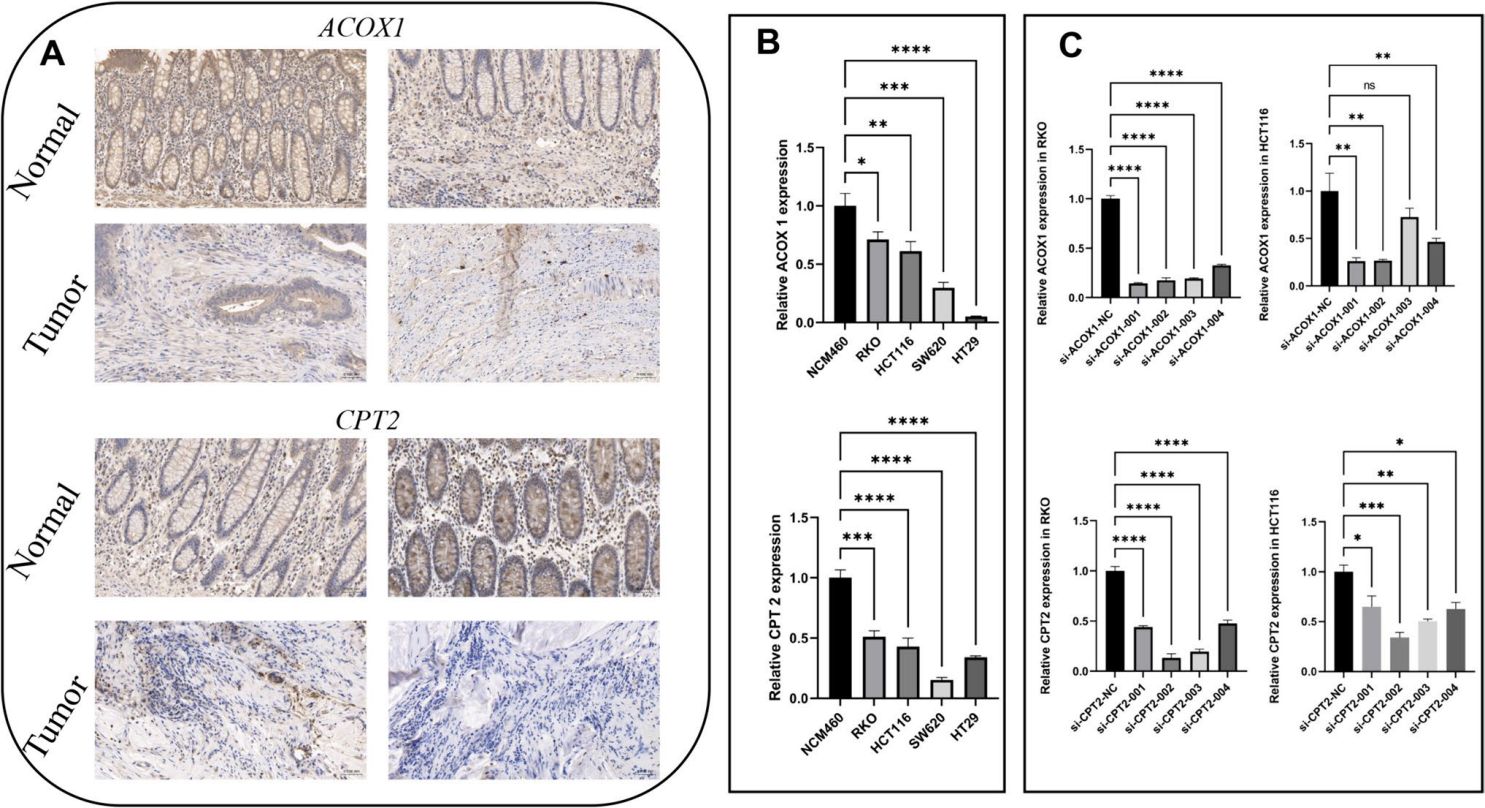
Expression of ACOX1 and CPT2 in CRC. (**A**) Immunohistochemical staining results showed the protein expression levels of ACOX1 and CPT2 in colorectal cancer tissues. (**B**) Compared with human intestinal epithelial NCM cell lines, ACOX1 and CPT2 were expressed at lower levels in CRC cell lines. (**C**) Relative expression of ACOX1 and CPT2 in CRC cells transfected with si-RNA or negative control (NC) was detected by RT-qPCR. ns, Not significant; * *p*< 0.05; ** *p*< 0.01; *** *p*< 0.001; **** *P*< 0.0001

We evaluated the expression of ACOX1 and CPT2 in 80 pairs of colorectal cancer tissues and adjacent normal tissues using IHC methods. Our results showed that ACOX1 and CPT2 were significantly downregulated in colorectal cancer tissues compared with normal tissues, as shown in Fig. 11A. Finally, we compared the expression levels of ACOX1 and CPT2 in normal intestinal epithelial cells and four CRC cell lines by PCR assay and found that the expression levels of ACOX1 and CPT2 genes were significantly down-regulated in tumor cells (Fig. 11B). These results strongly support the potential of ACOX1 and CPT2 as biomarkers for CRC diagnosis and prognosis. Subsequently, the expression levels of ACOX1 and CPT2 were evaluated after 5 days of transfection using qRT-PCR to validate the effect of siRNA knockdown of ACOX1 and CPT2 in RKO and HCT116 cell lines (Fig. 11C). Based on the knockdown efficiency, we chose the No. 1 and No. 2 siRNA knockdown cell lines for ACOX1-related functional experiments, whereas the No. 2 and No. 3 siRNA knockdown cell lines for CPT2-related functional experiments.

**Fig. 12:**
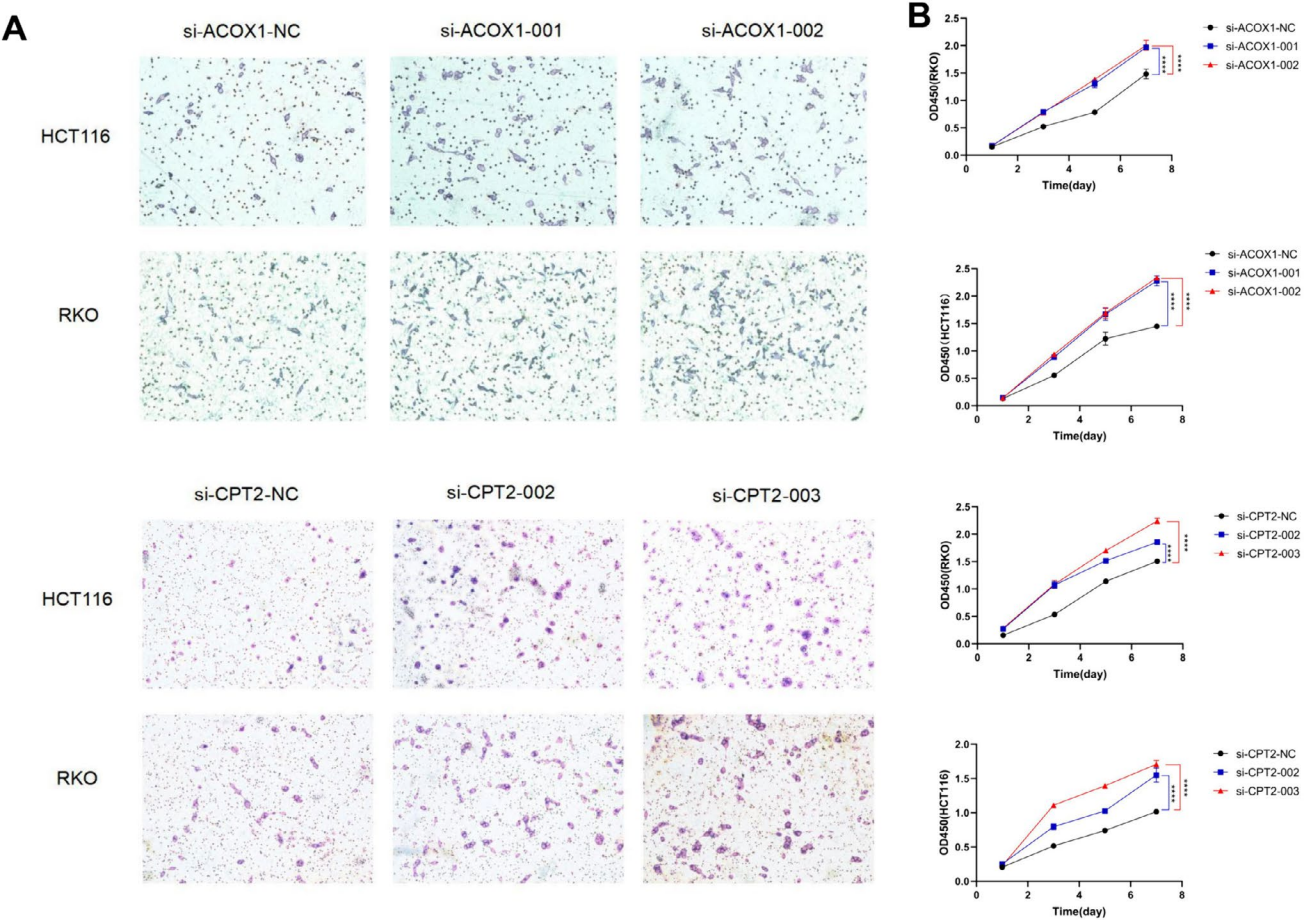
Functional experiments of ACOX1 and CPT2 in CRC. (**A**) Transwell assay showed that down-regulation of ACOX1 and CPT2 expression promoted the migration and invasion ability of CRC cells. (**B**) CCK8 assay showed that the proliferation ability of CRC cells with reduced expression of ACOX1 and CPT2 was significantly enhanced compared with the NC group. ns, Not significant; * *p*< 0.05; ** *p*< 0.01; *** *p*< 0.001; **** *P*< 0.0001

Subsequently, CCK-8 cell experiments showed that the knockdown-induced reduction of ACOX1 and CPT2 significantly enhanced the proliferation of RKO and HCT116 cell lines (Fig. 12B). Tumor cells transfected with si-ACOX1 and si-CPT2 also exhibited enhanced migration and invasion in transwell assays (Fig. 12A). Together, these findings suggest that ACOX1 and CPT2 are oncogenes for colorectal cancer.

## Discussion

Nucleotide metabolism is central to tumor development, with increased synthesis of NTPs and dNTPs in tumor cells (Ma et al. 2021). This metabolic feature, a potential therapeutic target, significantly affects cancer cell behavior, including proliferation, immune evasion, metastasis, and resistance to therapy. The elevated nucleotide levels are essential for DNA replication, repair, transcription, ribosome biosynthesis, and post-translational protein glycosylation—processes often dysregulated in cancer (Xia et al. 2021). Oncogenic driver genes, known to upregulate nucleotide biosynthesis, emphasize the metabolic phenotype's role in cancer initiation and progression post-oncogene activation. The relationship between NUDT5 and nucleotide metabolism in cancer is particularly noteworthy. NUDT5, an ADP-ribose pyrophosphatase, is vital for hydrolyzing ADP to regulate cellular functions, influencing key cellular processes. Zhang et al. found that reduced NUDT5 expression in HeLa cells leads to G1 cell cycle arrest and induces apoptosis in fibroblasts and RNA oxidation (Zhang et al. 2021c).

Nucleotide synthesis in cancer cells occurs through two main pathways: the de novo and recycling pathways (Koundinya et al. 2018; Wang et al. 2019). The de novo pathway involves a series of energy-intensive enzymatic reactions that convert small precursors into nucleotides. Conversely, the recycling pathway efficiently converts nucleosides or nucleobases into NMPs via phosphorylation or phosphoribosyltransferase reactions, with fewer steps. The unique mechanisms of purine and pyrimidine nucleotide synthesis in tumor cells are key for evading inhibitors targeting de novo synthesis. Early anti-tumor agents included nucleotide synthesis inhibitors, which remain crucial in cancer treatment (Dutta et al. 2023). In therapy-resistant tumors, targeting nucleotide synthesis pathways beyond one-carbon metabolism has proven effective. For example, 5-FU is fundamental in treating advanced colorectal cancer, and gemcitabine is used in pancreatic cancer, both by disrupting nucleotide synthesis to counter resistance mechanisms (Panieri and Santoro 2016). Lately, nucleotide synthesis has become a focal point in cancer research, frequently identified as a cancer cell vulnerability in extensive genomics, chemical screens, and metabolomics studies. This renewed interest is propelling impactful preclinical research, laying the groundwork for current clinical trials and drug development (Shi et al. 2023).

Single-cell sequencing and spatial transcriptomics are essential tools in colorectal cancer research, providing detailed insights at the cellular level. Single-cell sequencing allows for in-depth analysis of transcriptomic and genomic profiles, illuminating tumor heterogeneity and immune cell dynamics (Aran 2023). The integration of m6A modification analysis with single-cell transcriptomics has recently revealed important cellular subpopulation regulators and improved treatment response prediction in CRC patients (Gao et al. 2022). Moreover, single-cell sequencing is vital for exploring the tumor microenvironment and enhancing our understanding of immunotherapy (Qi et al. 2022). When paired with spatial transcriptomic techniques, these methods facilitate the study of cell-cell interactions across tissue, offering a comprehensive view of cellular spatial distribution and functionality within tumor contexts (Zhang et al. 2022). The progress in these technologies has greatly deepened our knowledge of CRC pathogenesis and is instrumental in advancing precision medicine and innovative therapeutic strategies.

Utilizing advanced single-cell sequencing and spatial transcriptomics, our study meticulously profiled nucleotide metabolism-related genes in colorectal cancer to differentiate between tumor and normal tissues. This approach aimed to elucidate the impact of nucleotide metabolism on CRC progression and its implications for immunotherapy. We identified considerable variability in gene expression at the single-cell level, revealing the complexity of nucleotide metabolism in CRC. Spatial transcriptomic analysis further revealed distinct expression patterns across cellular subpopulations and microenvironments. The heterogeneity observed suggests that diverse cell types may profoundly affect nucleotide synthesis and function, highlighting nucleotide metabolism as a promising therapeutic target in CRC.

Colorectal cancer samples exhibited significantly higher expression levels of genes related to nucleotide metabolism compared to adjacent cell types. This suggests elevated nucleotide metabolic activity within tumor tissues, as nucleotides and deoxyribonucleotides serve as substrates for cell growth and proliferation, with altered metabolism potentially associated with nucleotide synthesis (Elia and Haigis 2021). Previous studies have demonstrated heightened activation of key enzymes involved in pyrimidine nucleotide synthesis and salvage pathways, such as thymidylate and thymidylate kinase, in tumor cells, particularly in metastatic tumors (Zhang et al. 2014). Integrating this nucleotide metabolism signature into the spatial transcriptome via deconvolution revealed elevated scores associated with nucleotide metabolic signatures at the core regions of colorectal cancer tumors. While our current findings do not conclusively establish heightened nucleotide metabolism levels at the core of colorectal cancer, there appears to be an association between metabolic communication and one-carbon metabolism within the tumor microenvironment and colorectal cancer development. Leveraging spatial transcriptomic techniques, we further integrated the expression profiles of nucleotide metabolism-related genes with specific cell types and tissue structures, revealing spatial heterogeneity in nucleotide metabolism within the colorectal cancer microenvironment (Han et al. 2021). Notably, our observations, both at the single-cell sequencing and spatial data levels, indicate robust cellular crosstalk between epithelial cells and fibroblasts, with the latter exhibiting higher scores in nucleotide metabolism. Prior research has elucidated how tumor cells communicate with fibroblasts via the release of exosomes, impacting tumor proliferation and invasiveness (Zheng et al. 2020). Additionally, the interaction between cancer cells and tumor-associated fibroblasts (CAFs) influences tumor growth and invasion (Beatty et al. 2011). In the tumor microenvironment, cellular communication between tumor cells and fibroblasts may involve the release and exchange of nucleotide metabolites, thereby affecting cell growth, differentiation, and metabolic status.

Nucleotide metabolism plays a pivotal role in immunosuppression in colorectal cancer, affecting immune cell function, infiltration, and immune checkpoint suppression, which facilitates tumor immune evasion. To tackle this, we created a new risk-scoring system that utilizes nucleotide metabolism-related biomarkers to predict therapy outcomes and stratify risk in CRC patients from TCGA and GEO datasets. Patients were categorized into high and low-risk groups based on their gene expression patterns, with those in the high-risk group showing worse prognoses, increased TMB, and more TIIC infiltration. Our signature's predictive power for patient survival was confirmed across both training and validation cohorts, establishing its potential as an independent prognostic indicator in CRC.

Our research indicates that high-risk group patients exhibit an immunosuppressive tumor microenvironment (TME) in colorectal cancer, which is characterized by weakened immune surveillance, reduced immune cell infiltration, an abundance of immunosuppressive cells, and disrupted immune checkpoint pathways, all of which promote tumor immune evasion and progression. The elevated TME scores in these patients are associated with increased risk, aligning with previous studies that have shown mesenchymal and immunological scores to rise with disease advancement, signaling a poor prognosis in CRC (Zhang et al. 2017). High-risk colon cancer patients also demonstrate increased activity of immune checkpoint molecules such as PD-1 and CTLA-4, which hinder T-cell function and their capacity to target cancer cells. This is further supported by enrichment analysis showing a propensity for tumor invasion and metastasis in high-risk individuals, linked to the deregulation of JAK-STAT and TNF pathways. These pathways exacerbate the inflammatory environment within the TME of high-risk patients, suppressing the generation of anti-tumor inflammatory factors and thereby promoting tumor growth and spread. This comprehensive analysis of the CRC TME underscores the complexity of immune interactions and their critical role in disease progression and patient outcomes.

Higher Tumor Mutational Burden (TMB) often leads to more tumor neoantigens, potentially enhancing immune response and making tumors more recognizable by the immune system, which could improve responses to immunotherapy (Zhang et al. 2023a). Microsatellite Instability-High (MSI-H), prevalent in colorectal cancer, indicates a DNA repair deficiency, increasing mutational load and tumor immunogenicity, with immunotherapy showing better outcomes in MSI-H CRC patients (Li et al. 2023). However, our study found that despite high TMB and MSI-H, the immunotherapy response in the high-risk CRC group was unexpectedly poor. We discovered a negative correlation between risk scores and positive immune checkpoint blockade (ICB) signals and a positive correlation with a suppressed tumor immune cycle. To gauge the immune status and guide therapy, we used "Tide" and "IPS” metrics. A high Tide level suggests a low immunotherapy response due to a weak immune attack on tumor cells, while a higher IPS score indicates a more active immune system, potentially leading to better immunotherapy outcomes. Our findings link lower risk scores to higher immunotherapy response rates and better prognoses. Even with high TMB or MSI-H, CRC patients might develop immune evasion strategies, such as increased immune checkpoint expression, reduced tumor antigen presentation, and immune cell suppression by tumor-associated macrophages and Tregs, which could limit immunotherapy efficacy. Combining chemotherapy with immunotherapy is a key strategy in CRC, with chemotherapy potentially boosting immunotherapy by reducing tumor load and immunosuppression. Our study's finding of increased chemotherapeutic sensitivity in the low-risk group suggests that combining cisplatin with immunotherapies could enhance treatment benefits, possibly by stimulating CD8+ T cells and strengthening the anti-cancer immune response.

Our study meticulously utilized single-cell sequencing to pinpoint ACOX1 and CPT2 as pivotal oncogenes in the nucleotide metabolism of colorectal cancer. Underexpression of ACOX1, observed in Oral Squamous Cell Carcinoma (OSCC) and linked to tumor growth inhibition, suggests its role in tumor suppression, supported by its association with tumor formation in breast and pancreatic cancers (Zhou et al. 2021; Shen et al. 2020). The stability of ACOX1 is governed by DUSP14, which, through dephosphorylation, enhances ACOX1 degradation, thereby potentially accelerating CRC progression. This degradation leads to increased palmitoylation of β-linker proteins, furthering CRC advancement (Zhang et al. 2023b). CPT2, essential for fatty acid metabolism, is significantly downregulated in CRC, contributing to stem cell characteristics and oxaliplatin resistance. This downregulation triggers higher ROS levels, activates the Wnt/β-cyclin pathway, and boosts glycolysis, enhancing CRC cell stemness and chemoresistance (Li et al. 2021; Liu et al. 2022b). Delving into ACOX1 and CPT2's regulatory mechanisms promises to illuminate CRC's pathogenesis and pave the way for innovative treatments. Our results indicate that CRC cells with higher ACOX1 and CPT2 expression tend to have better prognoses and increased sensitivity to immunotherapy. Knockdown experiments reinforced that diminished ACOX1 and CPT2 expression contribute to malignancy, influencing proliferation, apoptosis evasion, and tumor invasiveness. In essence, our research emphasizes ACOX1 and CPT2's crucial roles in CRC development and their potential as therapeutic targets.

Our study conducted a comprehensive analysis of nucleotide metabolism gene expressions in colorectal cancer using single-cell sequencing and spatial transcriptomics. It revealed expression disparities among cellular subpopulations, underscoring nucleotide metabolism's role in tumor immune evasion and drug resistance. Focused on these gene expressions, we developed a risk score model predictive of CRC patient survival and associated with immunotherapy and chemotherapy responses, gene mutations, and the tumor microenvironment. The study also identified ACOX1 and CPT2 as significant prognostic markers in CRC, suggesting their potential as targets for immunotherapy.

## Conclusion

This study utilized single-cell sequencing and spatial transcriptomics to analyze nucleotide metabolism-related gene expressions in colorectal cancer, uncovering cellular subpopulation-specific variations. The research elucidated the intricate relationship between nucleotide metabolism, CRC's immune evasion, and drug resistance, leading to the creation of a predictive risk score model for patient survival. This model correlates with immunotherapy response, chemotherapy effectiveness, gene mutations, and the tumor microenvironment, highlighting its clinical significance. Additionally, ACOX1 and CPT2 were identified as potential prognostic indicators for CRC, indicating their potential as therapeutic targets. These insights advance our knowledge of CRC pathogenesis and could guide the development of tailored treatment approaches.

### Supplementary information

ESM 1 (XLS 365 kb)

ESM 2 (DOC 4787 kb)

## Data Availability

No datasets were generated or analysed during the current study.
