# Supplementary material for: Targeting nucleotide metabolic pathways in colorectal cancer by integrating scRNA-seq, spatial transcriptome, and bulk RNA-seq data
Source: Funct Integr Genomics. 2024 Apr 10;24(2):72. doi: 10.1007/s10142-024-01356-5 (PMC11004054; doi:10.1007/s10142-024-01356-5)
Supplement: Supplementary file 2 — (DOC 4787 kb) [file 10142_2024_1356_MOESM2_ESM.doc]

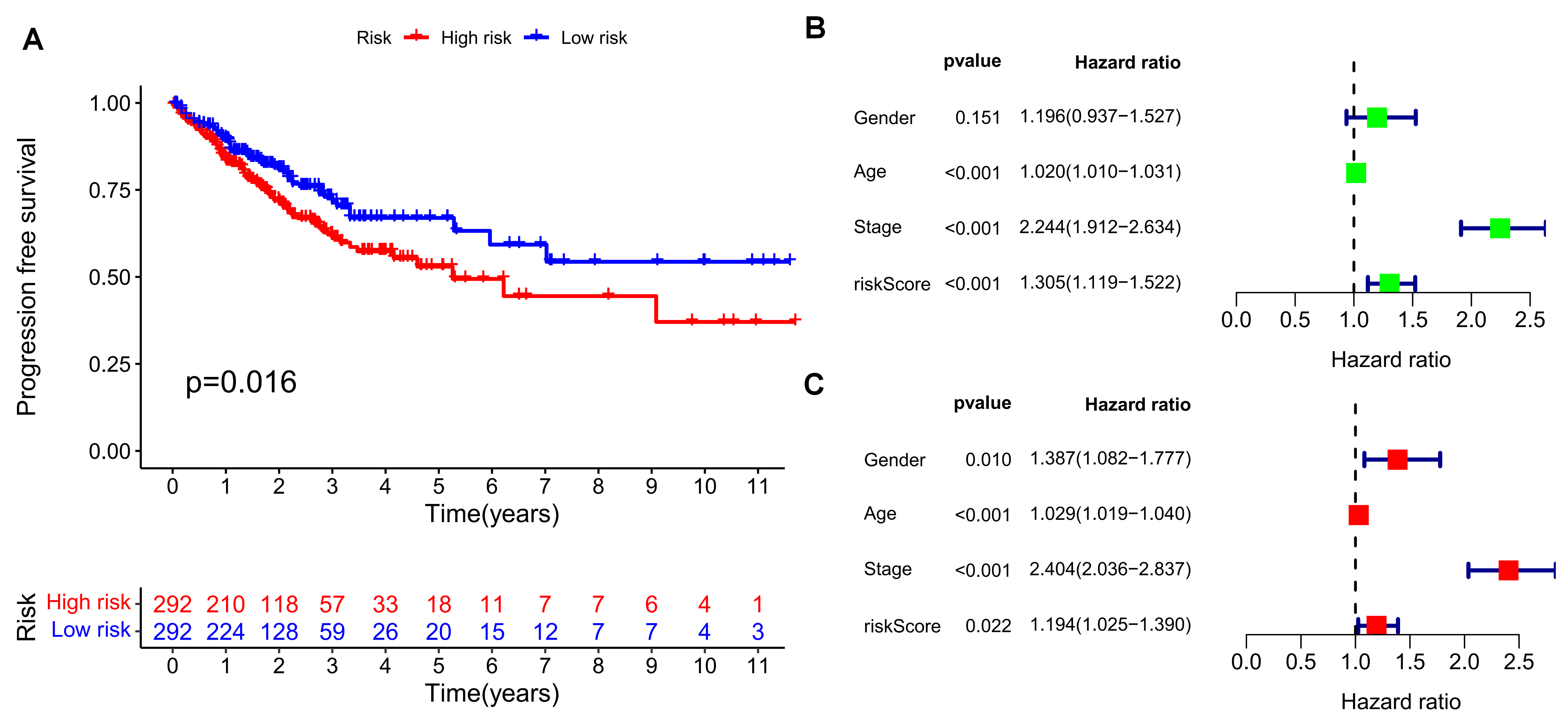


***Supplementary Figure 1.*** *(A) Progression-free survival time (PFS) of patients in different risk groups in the TCGA training set. (B,C) Univariate and multivariate Cox regression analyses of clinicopathologic variables and risk scores across the GEO validation set.*


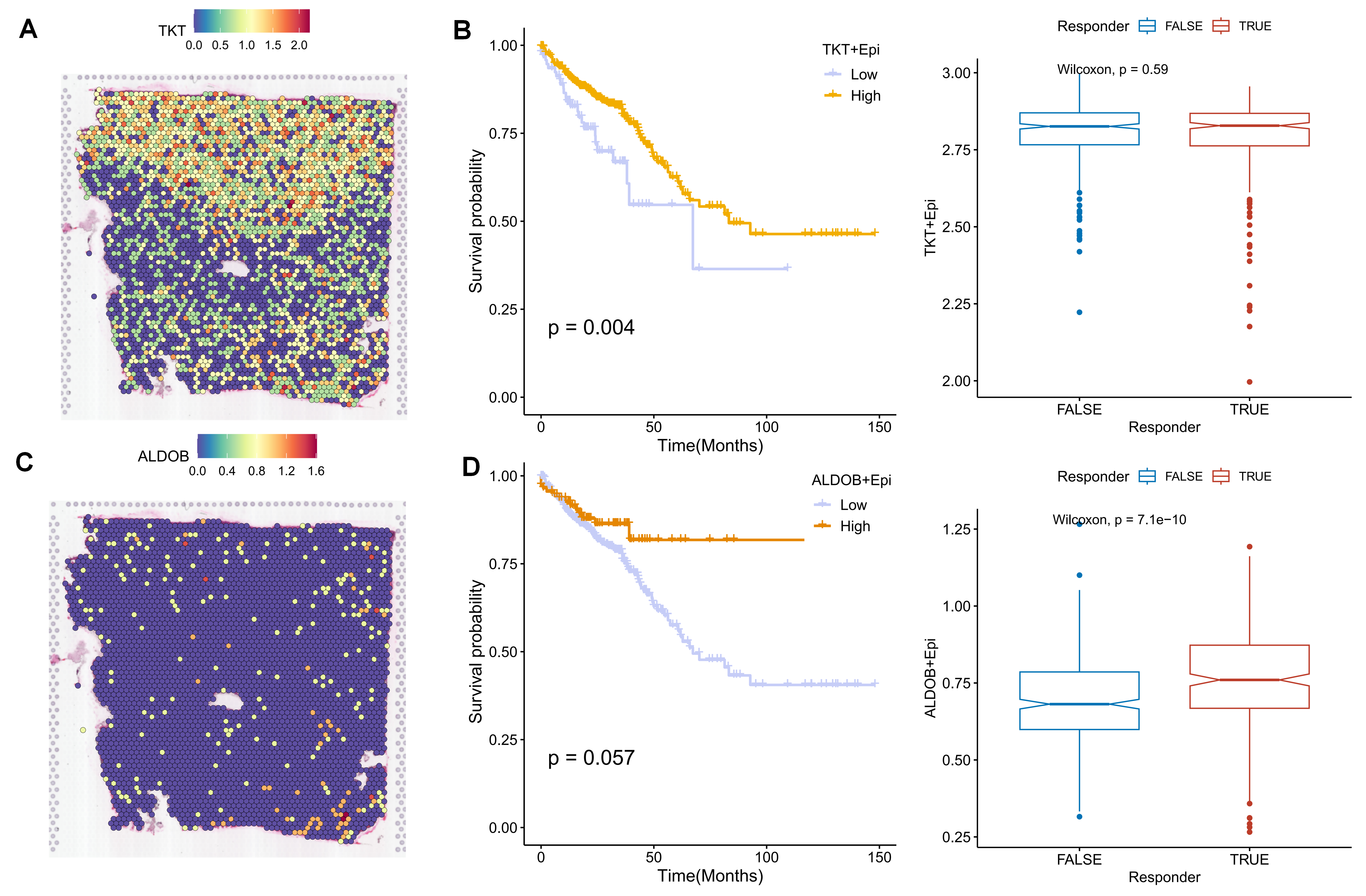


***Supplementary Figure 2.*** *(A) Spatial map demonstrating the expression of TKT in colorectal cancer. (B) Kaplan-Meier survival curves of OS for patients in the high and low TKT+ epithelial cell expression groups. Proportion of TKT+ epithelial cells in patients producing different immune responses. (C) Spatial plot demonstrating the expression of ALDOB in colorectal cancer. (D) Kaplan-Meier survival curves for OS in patients in the ALDOB+ epithelial cells high and low expression groups. Proportion of ALDOB+ epithelial cells in patients producing different immune responses.*
